# A missing enzyme-rescue metabolite as cause of a rare skeletal dysplasia

**DOI:** 10.1038/s41586-025-09397-x

**Published:** 2025-08-20

**Authors:** Jean Jacobs, Hristiana Lyubenova, Sven Potelle, Johannes Kopp, Isabelle Gerin, Wing Lee Chan, Miguel Rodriguez de los Santos, Wiebke Hülsemann, Martin A. Mensah, Valérie Cormier-Daire, Marieke Joosten, Hennie T. Bruggenwirth, Kyra E. Stuurman, Valancy Miranda, Philippe M. Campeau, Lars Wittler, Julie Graff, Stefan Mundlos, Daniel M. Ibrahim, Emile Van Schaftingen, Björn Fischer-Zirnsak, Uwe Kornak, Nadja Ehmke, Guido T. Bommer

**Affiliations:** 1 de Duve Institute-Biochemistry, UCLouvain, Brussels, Belgium; 2 Institute of Medical Genetics and Human Genetics, Charité—Universitätsmedizin Berlin, corporate member of Freie Universität Berlin and Humboldt–Universität zu Berlin, Berlin, Germany; 3 Max Planck Institute for Molecular Genetics, Berlin, Germany; 4 Institute of Chemistry and Biochemistry, Department of Biology, Chemistry and Pharmacy, Freie Universität Berlin, Berlin, Germany; 5 Julius Wolff Institute of Biomechanics and Musculoskeletal Regeneration, Charité Universitätsmedizin Berlin, Berlin, Germany; 6 BIH Center for Regenerative Therapies, Berlin Institute of Health at Charité Universitätsmedizin, Berlin, Germany; 7 Icahn School of Medicine at Mount Sinai, New York, NY USA; 8 Hand Surgery Department, Children’s Hospital Wilhelmstift, Hamburg, Germany; 9 Medical School Berlin, Berlin, Germany; 10 Department of Human Genetics, Helios Klinikum Berlin-Buch, Berlin, Germany; 11 BIH Biomedical Innovation Academy, Digital Clinician Scientist Program, Berlin Institute of Health at Charité Universitätsmedizin Berlin, Berlin, Germany; 12 Paris Cité University, Reference Center for Skeletal Dysplasia, INSERM UMR1163, Necker Enfants Malades Hospital, Imagine Institute, Paris, France; 13 Department of Clinical Genetics, Erasmus University Medical Center, Rotterdam, The Netherlands; 14 Division of Medical Genetics, Department of Pediatrics, CHU Sainte-Justine, Montreal, Quebec Canada; 15 German Center for Child and Adolescent Health (DZKJ), partner site Berlin, Berlin, Germany; 16 Institute of Human Genetics, University Medical Center Göttingen, Göttingen, Germany; 17 BIH Biomedical Innovation Academy, Clinician Scientist Program, Berlin Institute of Health at Charité Universitätsmedizin Berlin, Berlin, Germany

**Keywords:** Glycobiology, Enzymes, Disease genetics, Experimental models of disease, Deoxy sugars

## Abstract

Living cells depend on an intricate network of chemical reactions catalysed by enzymes, which sometimes make mistakes that lead to their inactivation. Here we report a metabolite-based mechanism for preserving enzyme function in an unfavourable environment. We found that the enzyme TGDS produces UDP-4-keto-6-deoxyglucose, a mimic of the reaction intermediate of the enzyme UXS1, which regenerates the essential cofactor NAD^+^ within the catalytic pocket of UXS1 by completing its catalytic cycle. Thus, the production of an ‘enzyme-rescue metabolite’ by TGDS represents a mechanism for maintaining the activity of an enzyme in a subcellular compartment where NAD^+^ is scarce. Using a combination of in vitro and in vivo studies, we demonstrate that the inability to produce sufficient amounts of this enzyme-rescue metabolite leads to the inactivation of UXS1, impairing the synthesis of specific glycans that are crucial for skeletal development. This provides an explanation for the development of the hereditary skeletal disorder Catel–Manzke syndrome in individuals with TGDS deficiency. Defects in similar protective layers might contribute to metabolic changes in other diseases that cannot be explained with common concepts in metabolic biochemistry.

## Abstract (web summary)

In mammals, the enzyme TGDS produces UDP-4-keto-6-deoxyglucose, which binds to the catalytic pocket of UDP-xylose synthase, thereby regenerating the essential NAD^+^ cofactor of UDP-xylose synthase in conditions of low NAD^+^.

## Main

Catel–Manzke syndrome (Online Mendelian Inheritance in Man (OMIM; https://omim.org/): 616145) is a rare skeletal dysplasia with variable clinical features including short stature, heart defects, micro- or retrognathia, cleft palate and malformations of the fingers^1–6^. It is caused by pathogenic variants in a gene encoding an enzyme of unknown function^2–6^, called dTDP-D-glucose 4,6-dehydratase (TGDS) owing to its similarity to bacterial enzymes that convert dTDP-glucose into dTDP-4-keto-6-deoxy-d-glucose in the synthesis of rhamnose during bacterial cell wall formation^7^. However, TGDS is likely to serve a different role in mammalian cells than in bacteria, as mammalian cells do not appear to produce dTDP-rhamnose^8^.

Two lines of evidence indicate that *TGDS* deficiency might be linked to a defect in the synthesis of glycosaminoglycans (GAGs), which have a key role in the extracellular matrix. First, Catel–Manzke syndrome shows clear phenotypic overlap with monogenic disorders of GAG metabolism, including the skeletal dysplasias Temtamy preaxial brachydactyly syndrome^1,9^ (OMIM: 605282), chondrodysplasia with joint dislocations, gPAPP type^10–12^ (linked to *IMPAD1* (also known as *BPNT2*); OMIM: 614078) and Desbuquois dysplasia 1 and 2^13,14^ (OMIM: 251450 and OMIM: 615777, respectively). Second, TGDS shows 25% amino acid identity with the enzyme UDP-xylose synthase (UXS1), which produces UDP-xylose, a nucleotide sugar that is required for the initial steps of GAG synthesis and the glycosylation of the protein α-dystroglycan^15,16^. In both mice and humans *UXS1* deficiency is associated with shortened long bones^17,18^.

## UXS1 is impaired in some *TGDS*-KO cells

**Fig. 1:**
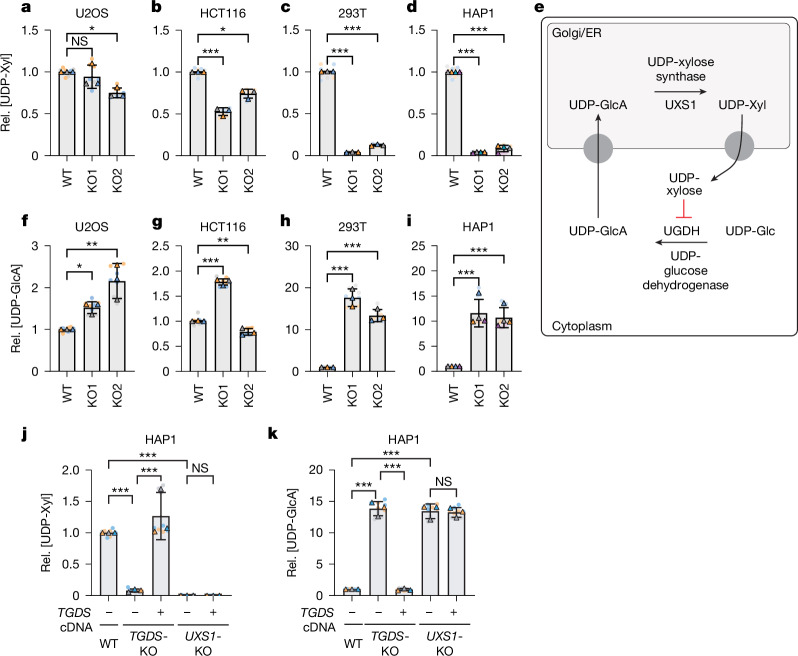
*TGDS* inactivation leads to a context-dependent inactivation of UXS1. **a**–**d**, UDP-xylose (UDP-Xyl) was measured by LC–MS in parental cells and two *TGDS-*KO clones generated in U2OS (**a**), HCT116 (**b**), 293T (**c**) or HAP1 (**d**) cells. Rel., relative. **e**, Schematic representation of the biosynthetic pathway of UDP-xylose. UXS1 produces UDP-xylose from UDP-glucuronate (UDP-GlcA) in the Golgi and endoplasmic reticulum (ER). In turn, UDP-xylose inhibits production of UDP-glucuronate by UDP-glucose dehydrogenase. UDP-Glc, UDP-glucose. **f**–**i**, UDP-glucuronate was quantified by LC–MS in the same cell lines as in **a**–**d**. **j**,**k**, UDP-xylose (**j**) and UDP-glucuronate (**k**) were quantified in parental, *TGDS-*KO and *UXS1*-KO HAP1 cells transduced with a lentivirus driving expression of TGDS or an empty control. Results support the hypothesis that loss of TGDS impairs UDP-xylose production and secondarily leads to accumulation of UDP-glucuronate. Data are normalized area under the curve for the indicated metabolites (mean ± s.d. of 3 (U2OS, HCT116, 293T) or 4 (HAP1) independent experiments, each containing 3 biological replicates) and are presented relative to wild-type (WT) control cell lines. Paired two-tailed Dunnett (**a**–**d**,**f**–**i**) or Sidak (**j**,**k**) post hoc testing of log-transformed data after one-way ANOVA. *P < 0.05, **P < 0.01, ***P < 0.001; NS, not significant. For exact *P* values see Source Data. Source data

**Extended Data Fig. 1:**
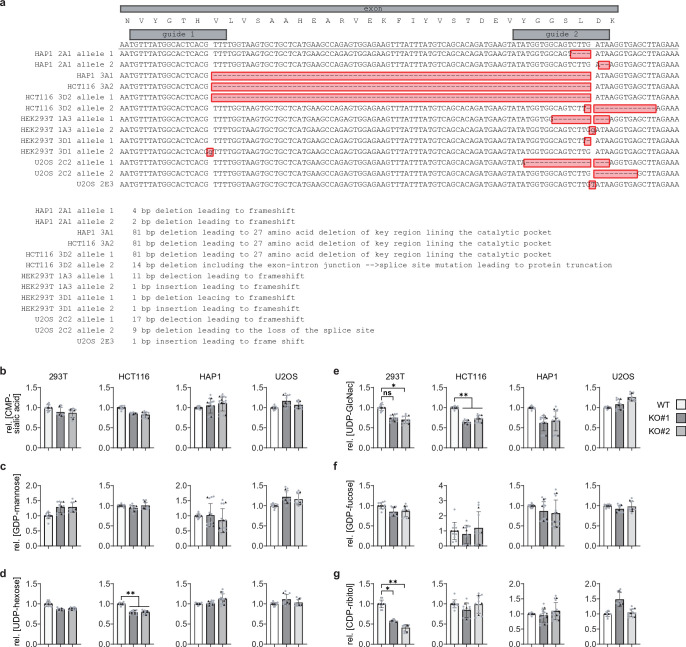
Inactivation of *TGDS* in 4 different cell lines does not affect most nucleotide sugar levels. **a**, Sequence of the genomic locus and predicted TGDS proteins in *TGDS*-deficient HAP1, U2OS, 293 T and HCT116 cell line clones. **b-g**, CMP-sialic acid (**b**), GDP-mannose (**c**), UDP-hexose (**d**), UDP-GlcNac (**e**), GDP-fucose (**f**), and CDP-ribitol (**g**) were determined by LC-MS in parental cells and two *TGDS* KO clones from U2OS, HCT116, HAP1 and 293 T. Data present means ± SD of three (U2OS, HCT116 and 293 T) or four (HAP1) experiments performed with 3 independent samples each, and are normalized to levels observed in wild-type cells. * p < 0.05 in paired two-tailed Dunnett *post-hoc* testing of log-transformed data after one-way ANOVA. For exact p-values see source data file. Source data

To gain insights into the molecular link between *TGDS* deficiency and GAG synthesis, we inactivated *TGDS* using CRISPR–Cas9 in a range of cell lines: the colorectal carcinoma cell line HCT116, the osteosarcoma cell line U2OS, the chronic myeloid lymphoma cell line HAP1 and the human embryonic kidney cell line 293T (Extended Data Fig. 1a). Next, we quantified nucleotide sugars using liquid chromatography–mass spectrometry (LC–MS), as they serve as direct biochemical precursors of GAGs. Most nucleotide sugars were unaffected by *TGDS* inactivation (Extended Data Fig. 1b–g). Yet, we observed approximately tenfold less UDP-xylose in *TGDS*-knockout (KO) 293T and HAP1 cells, whereas such reduction was largely absent when *TGDS* was inactivated in HCT116 and U2OS cells (Fig. 1a–d).

UDP-xylose is synthesized from UDP-glucuronate by the enzyme UXS1 in the endoplasmic reticulum and the Golgi apparatus. UDP-glucuronate itself is produced in the cytoplasm by UDP-glucose dehydrogenase (UGDH), an enzyme that is subject to feedback inhibition by UDP-xylose (Fig. 1e). A deficiency in UDP-xylose is expected to relieve this inhibition and result in increased UDP-glucuronate levels. Supporting this notion, we observed an up to 15-fold increase in UDP-glucuronate in cell lines in which *TGDS* knockout led to reduced UDP-xylose levels (HAP1 and 293T), whereas changes were more subtle or absent in cell lines in which UDP-xylose levels were maintained following *TGDS* knockout (HCT116 and U2OS) (Fig. 1f–i).

**Extended Data Fig. 2:**
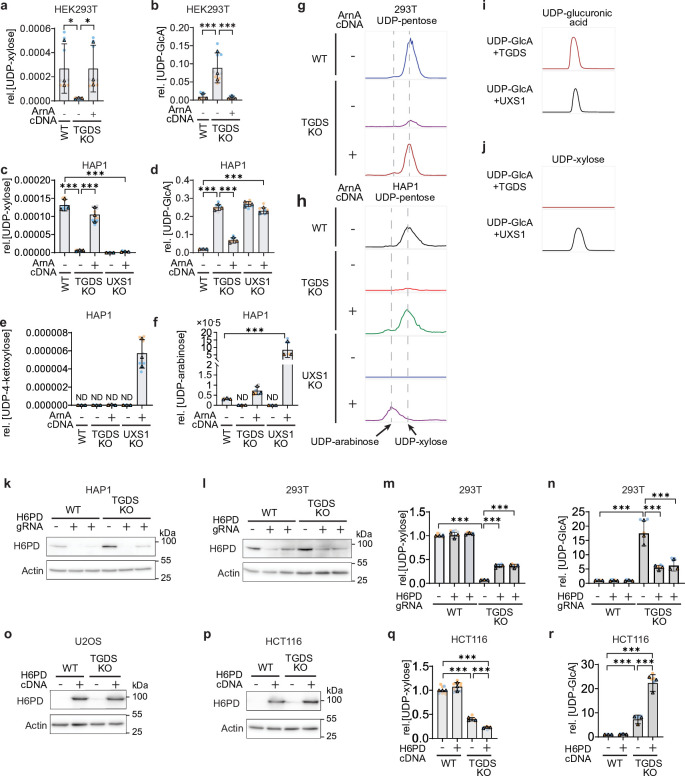
Data complementing Fig. 2. **a-f**, UDP-xylose (**a**,**c**), UDP-glucuronate (UDP-GlcA, **b**,**d**), UDP-4-ketoxylose (**e**) and UDP-arabinose (**f**) levels were determined by LC-MS in parental 293 T (**a**,**b**) and HAP1 (**c**-f) cells and in a *TGDS* KO or a *UXS*1 KO clone upon transduction with a recombinant lentivirus driving expression of ArnA (‘+’) or an empty vector control (‘-’). To allow comparison of the signal intensities obtained for different metabolites, data were only normalized to total ion current but not to the mean of the values observed in the parental cells within each experiment. **g-h**, Extracted ion chromatograms of the m/z of UDP-pentose (m/z 535.037) corresponding to panel **a** (**g**) and panels **c**,**f** (**h**). **i-j**, Extracted ion chromatograms of the m/z of UDP-GlcA (**i**, 579.027) or UDP-xylose (**j**, 535.037) in reactions containing 500 µM UDP-GlcA in the presence of 0.8 µM TGDS or 0.65 µM UXS1 for 24 h. **k-l**, Western blot for H6PD in wild-type and *TGDS*-deficient HAP1 (**k**) or 293 T (**l**) cell lines, transduced with lentiviral CRISPR/Cas9 constructs targeting *H6PD* (each ‘+‘ representing a different guide RNA) or a control gene (‘-‘), performed twice. See Supplementary Fig. 1 for uncropped images. **m-n**, UDP-xylose (**m**) and UDP-GlcA (**n**) were quantified in 293 T cell lines described in panel l. **o-p**, Western blot for H6PD in wild-type and a *TGDS*-deficient U2OS (**o**) or HCT116 (**p**) cell lines, transduced with recombinant lentiviruses driving expression of human *H6PD* cDNA (‘+‘) or an empty control (‘-‘), performed twice. **q-r**, UDP-xylose (**q**) and UDP-GlcA (**r**) were quantified in HCT116 cell lines described in panel **p**. Data are means ± SD of 3 independent experiments. * p < 0.05; ** p < 0.01; *** p < 0.001 in paired Sidak *post-hoc* testing of log-transformed data after one-way ANOVA. For exact p-values see source data file. Source data

To confirm that *TGDS* inactivation caused the observed effects, we restored its expression in the *TGDS-*KO cell lines, leading to a recovery of UDP-xylose levels and a decrease in UDP-glucuronate levels (Fig. 1j,k). Given that TGDS shares 25% amino acid identity with UXS1, we tested whether it could synthesize UDP-xylose by overexpressing it in *UXS1*-deficient cell lines. We did not observe any change in the amount of UDP-xylose, demonstrating that TGDS does not directly produce UDP-xylose (Fig. 1j and Extended Data Fig. 2i,j). Collectively, our results demonstrate that the inactivation of *TGDS* leads to a cell-type-specific functional deficiency of UXS1.

## A UDP-4-keto sugar can reactivate UXS1

**Fig. 2:**
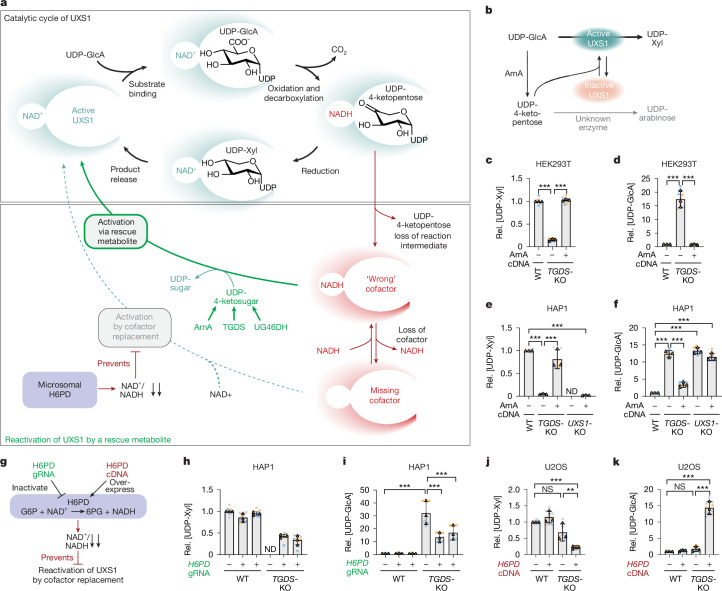
Functionally inactivated UXS1 requires reactivation by a UDP-4-keto sugar when H6PD is highly active. **a**, Working hypothesis: an abortive catalytic cycle of UXS1 leads to the inactivation of the enzyme, which is counteracted by UDP-4-keto sugars. The decarboxylation of UDP-glucuronate by UXS1 depends on the oxidation of the C4 hydroxyl group using a tightly bound NAD^+^. This generates NADH and a UDP-4-ketoxylose intermediate. Normally, formation of UDP-xylose regenerates NAD^+^, preparing the enzyme for another cycle. Infrequently, the intermediate dissociates from the catalytic pocket, leaving UXS1 bound to NADH and inactive. We hypothesized that UDP-4-keto sugars restore activity by facilitating the oxidation of enzyme-bound NADH to NAD^+^. Loss of the cofactor can also yield an inactive apoenzyme, but reactivation by NAD^+^ binding is limited by the low NAD^+^/NADH ratio in the endoplasmic reticulum maintained by H6PD. **b**, Schematic of the experiment to test whether UDP-4-ketoxylose produced by ArnA can reactivate UXS1. **c**–**f**, ArnA expression rescues UDP-xylose synthesis in *TGDS-*KO cells, but not in *UXS1*-KO cells. UDP-xylose (**c**,**e**), UDP-glucuronate (**d**,**f**) were quantified by LC–MS in parental 293T (**c**,**d**) and HAP1 (**e**,**f**) cells, and in *TGDS-*KO or *UXS1*-KO clones transduced with a lentivirus expressing ArnA or an empty vector. ND, not determined. **g**, Experiment exploring the role of H6PD in UXS1 dependency on TGDS. **h**–**k**, H6PD modulates UXS1 dependency on TGDS. In HAP1 cells, CRISPR–Cas9-mediated *H6PD* knockdown (using two different guide RNAs (gRNAs) versus control gRNA (−)) reduces the effect of *TGDS* deficiency on UXS1 function (**h**,**i**). In U2OS cells, *H6PD* overexpression (+) makes UXS1 activity dependent on TGDS (**j**,**k**). −, empty vector control. UDP-xylose (**h**,**j**) and UDP-glucuronate (**i**,**k**) were quantified by LC–MS. Data are mean ± s.d. of 3 independent experiments, each containing 3 biological replicates. Paired two-tailed Dunnett (**c**,**d**) or Sidak (**e**,**f**,**i**–**k**) post hoc testing of log-transformed data after one-way ANOVA. For exact *P* values see Source Data. Source data

The catalytic mechanism of UXS1 suggested how this enzyme might be inactivated. UXS1 catalyses the synthesis of UDP-xylose in two steps (Fig. 2a). First, the hydroxyl group on carbon 4 of UDP-glucuronate is oxidized using an enzyme-bound NAD^+^ as electron acceptor^19^. This strongly favours the decarboxylation yielding a UDP-4-ketoxylose reaction intermediate and a reduced cofactor NADH bound to the catalytic pocket. In the second step, the 4-keto group is reduced resulting in UDP-xylose and the restoration of the enzyme to its NAD^+^-bound form. Previous studies have shown that UXS1 may sometimes lose the UDP-4-ketoxylose intermediate and/or its cofactor, leaving the enzyme bound to its reduced cofactor NADH or as apoenzyme^20,21^ (Fig. 2a).

On the basis of studies with related enzymes, we expected that NADH-bound UXS1, which formed upon loss of the reaction intermediate, would be inactive^22–24^, but might be reactivated by a UDP-4-keto sugar. To test this, we used the bacterial enzyme ArnA, which catalyses the conversion of UDP-glucuronate to UDP-4-ketoxylose, but only produces negligible amounts of UDP-xylose^20^. Given that UDP-4-ketoxylose is the physiological reaction intermediate of UXS1, we reasoned that this metabolite should also restore the function of UXS1 (Fig. 2b). Overexpression of the bacterial enzyme ArnA in 293T and HAP1 *TGDS-*KO cells resulted in the recovery of UDP-xylose levels and a normalization of UDP-glucuronate levels (Fig. 2c–f). This effect was not observed in *UXS1*-deficient HAP1 cells, demonstrating that UDP-xylose was indeed synthesized by UXS1 (Fig. 2e,f). Notably, overexpression of ArnA also led to an increase in UDP-arabinose, particularly in *UXS1*-deficient cells, indicating that UDP-4-ketoxylose is reduced not only to UDP-xylose during the reactivation of UXS1 but also, by an unknown enzyme, to form UDP-arabinose (Extended Data Fig. 2a–h).

Overall, our data support a model in which UXS1 becomes functionally inactivated in *TGDS-*KO cells but can be reactivated by a UDP-4-keto sugar (Fig. 2a).

## H6PD makes UXS1 activity dependent on TGDS

**Extended Data Fig. 3:**
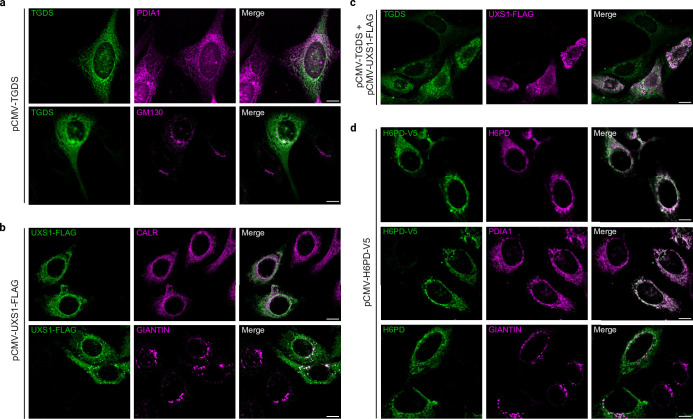
TGDS and UXS1 localize to the ER and the Golgi apparatus, whereas H6PD is limited to the ER. Immunofluorescence images of U2OS cells after transfection with pCMV-TGDS, pCMV-UXS1-FLAG and pCMV-H6PD-V5. TGDS localization was detected using anti-TGDS, UXS1 using anti-FLAG, and H6PD using either anti-H6PD or anti-V5 antibodies. Endogenous markers for the ER (PDIA1; CALR) and Golgi (GM130; GIANTIN) were used to determine the proteins localization. All panels except the costaining with GM130 (i.e. bottom three images in panel **a**) were performed with methanol-fixed cells. In the latter case, paraformaldehyde fixation was needed, which explains the slightly different morphology of the TGDS expression pattern. Scale bars, 10 µm.

The release of both the 4-keto intermediate and NADH from the catalytic pocket results in an inactive apoenzyme, which could be reactivated through the binding of NAD^+^^20,21^ (Fig. 2a, dashed arrow). This raised the question of why some cell lines (for example, HAP1 and 293T) depend on TGDS to maintain UXS1 activity, rather than simply recruiting a new NAD^+^ cofactor. We hypothesized that the subcellular localization of UXS1 and TGDS in the endoplasmic reticulum and the Golgi apparatus might underlie this dependence (Extended Data Fig. 3a–c). The microsomal enzyme hexose-6-phosphate dehydrogenase (H6PD) (Extended Data Fig. 3d) is known to maintain a very low NADP^+^/NADPH ratio in the endoplasmic reticulum to support the function of reductases^25,26^. It is a bifunctional enzyme that catalyses the two steps required for the oxidation of glucose-6-phosphate to 6-phosphogluconate. Notably, H6PD can also utilize NAD^+^ as a cofactor^27^. Consequently, the NAD^+^/NADH ratio in the endoplasmic reticulum and the Golgi apparatus is also expected to be very low^28^. As a result, NAD^+^ levels might be insufficient to support UXS1 reactivation in the absence of TGDS.

To test this, we inactivated *H6PD* using a lentiviral CRISPR–Cas9 approach in cell lines that depend on TGDS for UXS1 activity (HAP1 and 293T). In *TGDS*-deficient lines, *H6PD* inactivation led to a significant increase in UDP-xylose levels and a corresponding decrease in UDP-glucuronate, consistent with the idea that these cells can reactivate UXS1 without TGDS when H6PD is absent (Fig. 2g–i and Extended Data Fig. 2k–n). We also performed the reverse experiment by overexpressing H6PD in cell lines that are less dependent on TGDS to maintain UXS1 activity (U2OS and HCT116). We reasoned that forced overexpression of H6PD would lower NAD^+^ levels preventing these cells from maintaining UXS1 activity in the absence of TGDS. Indeed, overexpression of H6PD in *TGDS*-deficient U2OS cells and, to a lesser extent, in HCT116 cells, led to a decrease in UDP-xylose and an increase in UDP-glucuronate levels (Fig. 2j,k and Extended Data Fig. 2o–r). Overall, this provides evidence that cell-type-specific differences in H6PD levels and activity strongly contribute to the differences in the dependence on TGDS to maintain UXS1 function.

## The TGDS product rescues UXS1 function

**Fig. 3:**
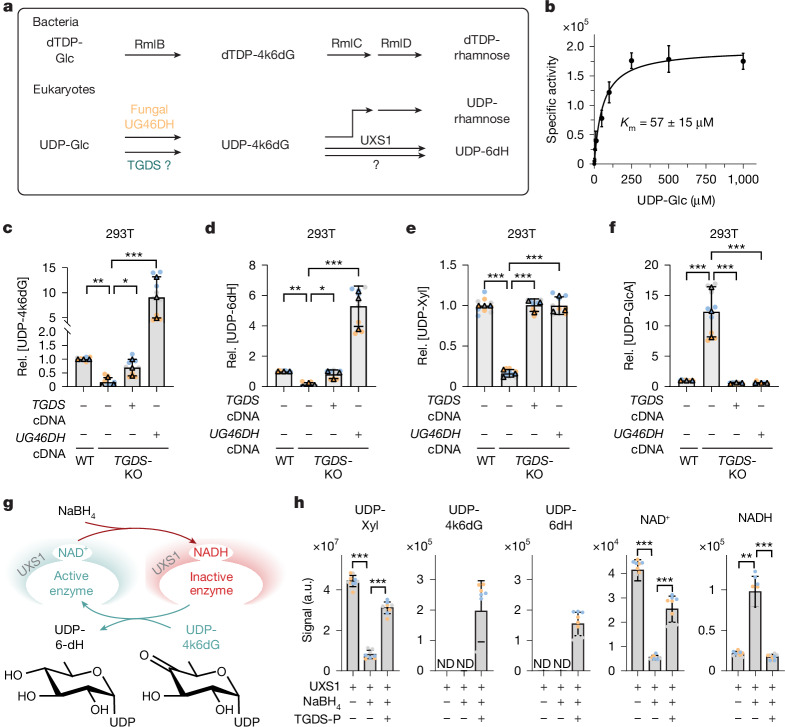
TGDS produces UDP-4-keto-6-deoxyglucose, an enzyme-rescue metabolite for UXS1. **a**, Schematic of the suggested UDP-glucose-4,6-dehydratase activity of TGDS (bottom), and UDP-rhamnose or dTDP-rhamnose synthesis in bacteria (top). UDP-6dH, UDP-6-deoxyhexose. 4k6dG, 4-keto-6-deoxyglucose. **b**, TGDS activity was assessed at the indicated concentrations of UDP-glucose for 4 h at 30 °C. Formation of UDP-4-keto-6-deoxyglucose is presented in arbitrary units. **c**–**f**, Expression of human TGDS or *B. cinerea* UG46DH rescue the phenotype of *TGDS-*KO cells. UDP-4-keto-6-deoxyglucose (**c**), UDP-6-deoxyhexose (**d**), UDP-xylose (**e**) and UDP-glucuronate (**f**) were quantified in parental and *TGDS-*KO 293T cells upon transduction with recombinant lentiviruses driving expression of human TGDS or *B. cinerea* UG46DH. Metabolite levels in *TGDS-*KO cells in **c**,**d** were close to background. Levels of UDP-4-keto-6-deoxyglucose and UDP-6-deoxyhexose in *TGDS*-KO cells were unaffected by the inactivation of H6PD (Extended Data Fig. 4i–l). **g**, Experimental setup to assess the reactivation of UXS1 via the product of TGDS. **h**, The TGDS product rescues functionally inactivated UXS1. UDP-xylose, UDP-4-keto-6-deoxyglucose, UDP-6-deoxyhexose, NAD^+^ and NADH were quantified by LC–MS in reactions where UDP-glucuronate was incubated with 0.56 µM UXS1, sodium borohydride-inactivated UXS1 or inactivated UXS1 in the presence of a 1.5-fold excess of the product of TGDS (TGDS-P). Data are mean ± s.d. from three independent experiments, each containing three biological replicates in **c**–**f**,**h**. Paired two-tailed Sidak (**c**–**f**) or Dunnett (**h**) post hoc testing of log-transformed data after one-way ANOVA. For exact *P* values see Source Data. Source data

**Extended Data Fig. 4:**
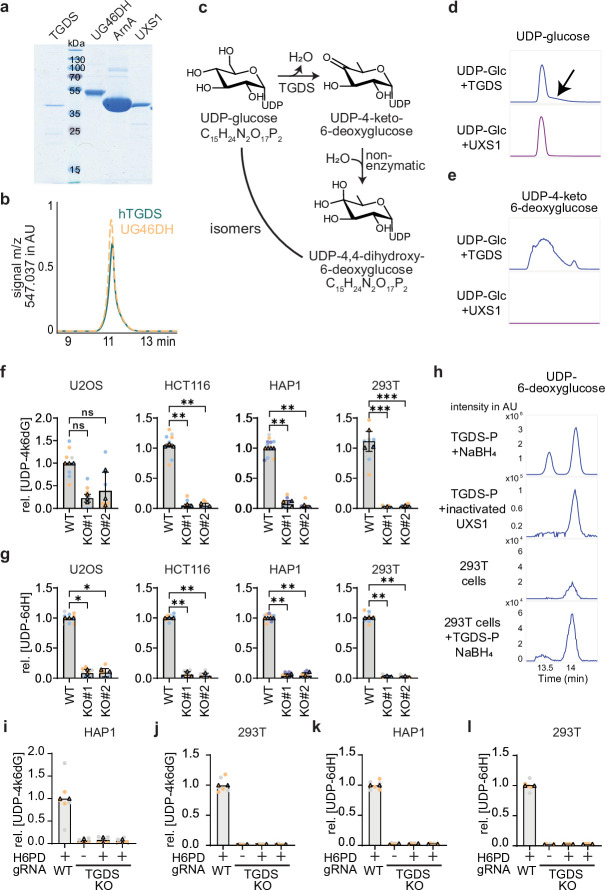
Levels of the TGDS product UDP-4-keto-6-deoxyglucose and of UDP-6-deoxyhexose are lower in all *TGDS* KO cells and unaffected by inactivation of H6PD. **a**, Recombinant purified human TGDS, *B. cinerea* UG46DH, *E. coli* ArnA and human UXS1 analysed by SDS-PAGE and Coomassie staining to demonstrate purity. **b**, Extracted ion chromatogram (EIC) of the m/z corresponding to UDP-4-keto-6-deoxyglucose (i.e., 547.037) in reactions of recombinant fungal UG46DH or human TGDS with UDP-glucose. **c**, UDP-4-keto-6-deoxyglucose, the reaction product of UG46DH and TGDS, is partially hydrated in a non-enzymatic reaction to form UDP-4,4-dihydroxy-6-deoxyglucose, which has the same mass as UDP-glucose^30^. **d-e**, Extracted ion chromatograms of the m/z of UDP-glucose (**d**, 565.048) and UDP-4-keto-6-deoxyglucose (**e**, UDP-4k6dG, 547.037) in reactions containing 500 µM UDP-glucose in the presence of 0.8 µM TGDS or 0.65 µM UXS1 for 24 h. The trailing peak (arrow, **d**) in the ‘UDP-glucose’ chromatogram corresponds to the hydrated form of UDP-4k6dG previously reported in reactions for fungal orthologues^30^. **f-g**, UDP 6-deoxyhexose (UDP-6dH, f) and UDP-4-keto-6-deoxyglucose (UDP-4k6dG, **g**) levels were determined by LC-MS in parental cells and 2 *TGDS* KO clones from U2OS, HCT116, HAP1 and 293 T. **h**, Tentative identification of UDP-6-deoxyhexose as UDP-6-deoxyglucose. Extracted ion chromatogram (EIC) of the m/z corresponding to UDP-6-deoxyhexose (i.e., 549.053) in a reaction where the TGDS product (TGDS-P) was chemically reduced with NaBH_4_, leading to the formation of UDP-6-deoxyglucose and UDP-6-deoxygalactose (upper panel). Based on the catalytic cycle of UXS1, only UDP-6-deoxyglucose formation is expected when the TGDS product is incubated with UXS1 that has been inactivated using NaBH_4_ beforehand (second panel). The UDP-6-deoxyhexose peak in cells coelutes with the one corresponding to UDP-6-deoxyglucose (third panel). Coelution is corroborated (lower panel) by the fact that the peak increases upon addition of a small amount of the sample presented in the upper panel. **i-l**, Inactivation of H6PD in *TGDS*-deficient cells does not lead to an increase in the rescue metabolite UDP-4k6dG (**i**,**j**) nor in UDP-6dH (**k**,**l**). LC-MS analysis for the indicated metabolites was performed in wild-type and a *TGDS*-deficient HAP1 (**i**,**k**) or 293 T (**j**,**l**) cell line, transduced with lentiviral CRISPR/Cas9 constructs targeting H6PD (each ‘+‘ representing a different guide RNA) or a control gene (‘-‘). Data are means ± SD of the means obtained in three (**f**,**g**) and two (**i**-**l**) independent experiments performed with three biological replicates. * p < 0.05; ** p < 0.01; *** p < 0.001 in paired two-tailed Dunnett (f-g) *post-hoc* testing of log-transformed data after one-way ANOVA. For exact p-values see source data file. Source data

The enzymatic function of human TGDS is unknown, but it shares 34% identity with RmlB from *E. coli*^29^ and 47% identity with UDP-glucose-4,6-dehydratase (UG46DH) from the fungus *Botrytis cinerea* (also known as *Botryotinia fuckeliana*)^30^, which catalyse dehydration reactions on dTDP-glucose and UDP-glucose, respectively (Fig. 3a). Since dTDP-glucose is not known to be present in mammalian cells, we focused on the UDP-glucose-4,6-dehydratase activity. We produced recombinant fungal UG46DH and human TGDS in *E. coli* (Extended Data Fig. 4a). Next, we incubated UDP-glucose with these enzymes and analysed the reactions by LC–MS. TGDS produced a metabolite with identical *m*/*z*, elution time and hydration status as UDP-4-keto-6-deoxyglucose produced by fungal UG46DH^30^ (Fig. 3b and Extended Data Fig. 4b–e). To our knowledge, this is the first enzymatic activity described for TGDS. This reaction has a Michaelis constant (*K*_M_) of approximately 60 µM with respect to UDP-glucose, thus we expect that its activity is saturated at concentrations of this substrate in the endoplasmic reticulum and the Golgi apparatus^31,32^. Together with a very low maximal velocity (*V*_max_) of 1.4 nmol min^−1^ mg^−1^, this should enable a continuous low-rate production of UDP-4-keto-6-deoxyglucose.

So far, UDP-4-keto-6-deoxyglucose has not been reported as a physiological metabolite in mammalian cells. Yet, we observed a signal for a metabolite with the same retention time and *m*/*z* in wild-type cells, which was undetectable in *TGDS-*KO cell lines, even in those where no effect on UDP-xylose had been detected (Fig. 3c and Extended Data Fig. 4f). Levels of this metabolite were tightly correlated with levels of another metabolite with a molecular weight corresponding to a UDP-6-deoxyhexose, tentatively identified as UDP-6-deoxyglucose (Fig. 3d and Extended Data Fig. 4g,h). This indicated that UDP-4-keto-6-deoxyglucose is partially reduced to this metabolite during the reactivation of UXS1 or via a hitherto unknown enzyme, similar to what we had observed for the product of ArnA (Fig. 3a and Extended Data Fig. 2f–h). Re-expression of TGDS or fungal UG46DH increased the amounts of both metabolites, reaching supraphysiological levels with the fungal enzyme (Fig. 3c,d). Both interventions restored UDP-xylose and decreased UDP-glucuronate levels in *TGDS-*KO cells (Fig. 3e,f), indicating that UDP-4-keto-6-deoxyglucose can reactivate UXS1. In parental cells, concentrations for this metabolite remained below 200 nM, corresponding to an estimated concentration of <1.5 µM in the endoplasmic reticulum and the Golgi apparatus^33^. This suggests that very low concentrations suffice to reactivate UXS1 in cells.

**Extended Data Fig. 5:**
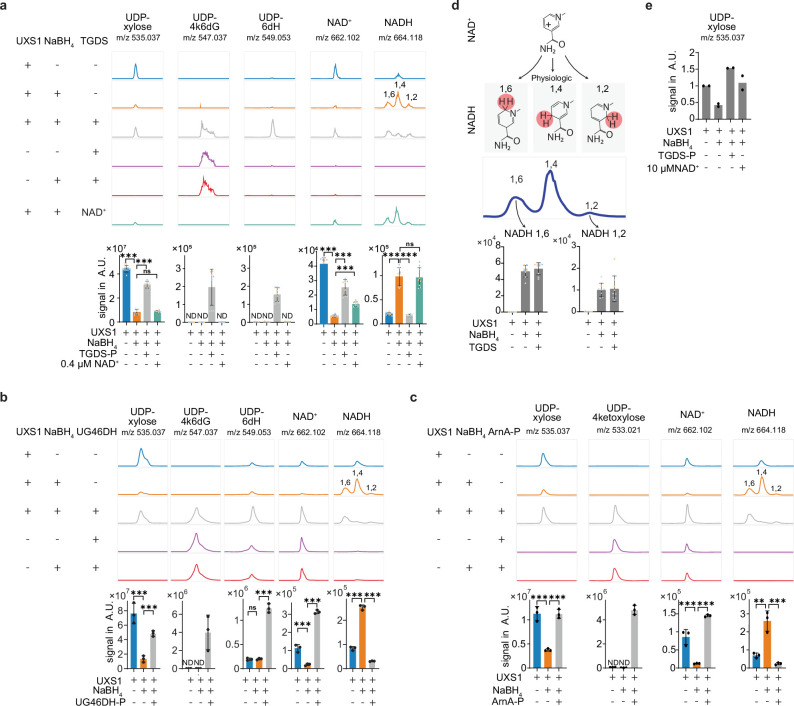
Products from the bacterial and fungal orthologue of TGDS reactivate inactivated UXS1. **a**, Extracted ion chromatograms for the m/z corresponding to UDP-xylose, UDP-4-keto-6-deoxyglucose (‘UDP-4k6dG’), UDP-6-deoxyhexose (‘UDP-6dH’), NAD^+^, and NADH, obtained by LC-MS in reactions where UDP-glucuronate was incubated with UXS1, with sodium-borohydride-inactivated UXS1, or with inactivated UXS1 in the presence of the product of human TGDS (‘TGDS-P’). Quantifications as in Fig. 3h, but including a condition where 0.4 µM NAD^+^ was added, demonstrating that low levels of free NAD^+^ present in the TGDS preparation do not suffice to rescue UDP-xylose production. **b-c**, Experiment described in panel **a** but using the *Botrytis cinerea* UG46DH product (‘UG46DH-P’, **b**) or *E. coli* ArnA product (‘ArnA-P’, **c**). **d**, Schematic representation of the formation of different NADH forms as well as the quantification of 1,6-NADH and 1,2-NADH in the experiment presented in Fig. 3h. **e**, Quantification of UDP-xylose production in an experiment as described in Fig. 3h, but including a condition where 10 µM NAD^+^ was added, demonstrating that UXS1 activity can be restored by sufficient amounts of NAD^+^. Data are means of two (e) or means ± SD of three independent experiments, each consisting of three (**a**,d) or one biological replicate (**b**,**c**). * p < 0.05, ** p < 0.01, *** p < 0.001 in two-tailed paired Dunnett *post-hoc* testing of log-transformed data after one-way ANOVA. For exact p-values see source data file. Source data

Next, we tested whether UDP-4-keto sugars could rescue UXS1 from inactivation in vitro at concentrations observed in cells. To do this, we incubated recombinant UXS1 with sodium borohydride, which has previously been shown to reduce NAD^+^ bound in the catalytic site of related enzymes^22^ (Fig. 3g). This led to tenfold lower levels of NAD^+^ and a concomitant increase in NADH. As expected, the chemical reduction of NAD^+^ led to the formation of not only the physiological 1,4-NADH, but also the two other isoforms 1,2-NADH and 1,6-NADH (Fig. 3h and Extended Data Fig. 5a–d). Upon incubation with UDP-glucuronate, production of UDP-xylose was approximately threefold lower than from the untreated enzyme, consistent with the partial inactivation of the enzyme (Fig. 3h and Extended Data Fig. 5a–c). Next, we incubated the enzyme with UDP-4-keto-6-deoxyglucose produced in vitro by human TGDS or fungal UG46DH, as well as UDP-4-ketoxylose produced by ArnA (Fig. 3h and Extended Data Fig. 5a–c). We observed an almost complete recovery of UDP-xylose production, an increase of NAD^+^, and a concomitant decrease in 1,4-NADH levels (Fig. 3h and Extended Data Fig. 5a–c). This decrease only concerned the physiological form, 1,4-NADH, whereas 1,2-NADH and 1,6-NADH were unaffected (Extended Data Fig. 5a–d), indicating that 1,4-NADH was reoxidized using UDP-4-keto-6-deoxyglucose via the catalytic action of UXS1, rather than via a direct chemical reaction. Of note, UXS1 activity was also rescued by NAD^+^ (Extended Data Fig. 5e), consistent with previous reports^20^ and our observation that some UXS1 activity can be maintained when *H6PD* is knocked out (Fig. 2h,i and Extended Data Fig. 2k–n).

**Fig. 4:**
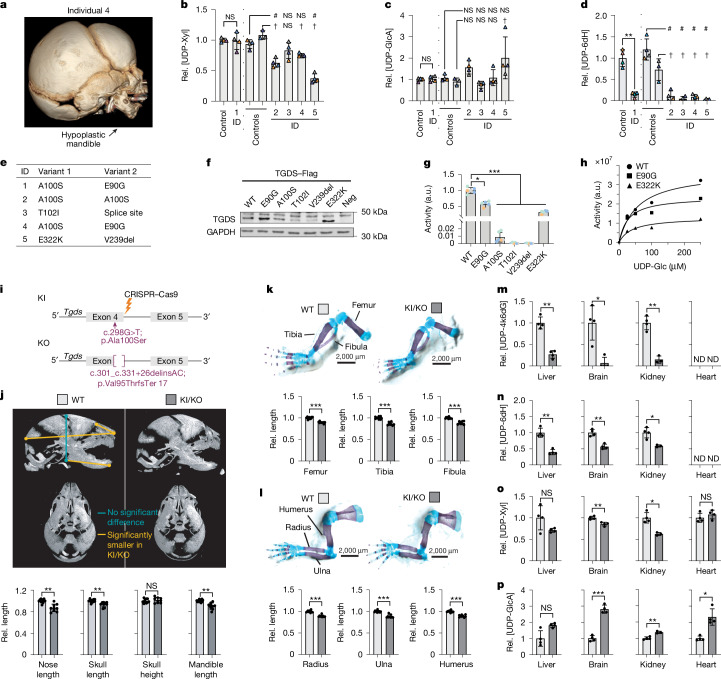
Patient-derived cell lines and a mouse model of Catel–Manzke syndrome corroborate the molecular function of *TGDS*. **a**, 3D reconstruction of the facial skeleton in individual 4. **b**–**d**, UDP-xylose (**b**), UDP-glucuronate (**c**) and UDP-6-deoxyhexose (**d**) were measured in fibroblasts from healthy controls and individuals with Catel–Manzke syndrome (ID1–5). Samples were analysed in two batches: individual 1 versus control 1; individuals 2–5 versus controls 2 and 3. **e**, Pathogenic variants of TGDS proteins. **f**, Western blot of Flag-tagged TGDS after transfection in U2OS cells. Quantification in Extended Data Fig. 6f and uncropped images in Supplementary Fig. 1. **g**,**h**, UDP-4-keto-6-deoxyglucose production by recombinant wild-type TGDS and indicated variants at 500 µM UDP-glucose for 24 h at 30 °C (**g**) or indicated UDP-glucose concentrations for 4 h at 30 °C (**h**). a.u., arbitrary units. **i**, Schematic of *Tgds*^*A100S/−*^ (KI/KO) mice carrying p.Ala100Ser and a frameshift deletion–insertion, causing loss of function. Created in BioRender. Lyubenova, H. (2025) https://BioRender.com/hsez57t. **j**, µCT sagittal and coronary images of E18.5 wild-type and KI/KO embryos showing brachycephaly, with shorter mandibles and snouts. Measurements that are significantly different between wild-type and KI/KO embryos are indicated in yellow. **k**,**l**, Skeletal preparations showing shortened hindlimb (**k**) and forelimb (**l**) long bones in KI/KO E18.5 embryos. **m**–**p**, Quantification of UDP-4-keto-6-deoxyglucose (**m**), UDP-6-deoxyhexose (**n**), UDP-xylose (**o**) and UDP-glucuronate (**p**) in organ lysates from 8-month-old wild-type and KI/KO mice. ND, not detectable for technical reasons. Data are mean of two (**h**) or mean ± s.d. from four (**b**–**d**,**g**) independent experiments; from 11 wild-type (**j**–**l**) and 9 KI/KO (**j**–**l**) mice; or from 4 wild-type and KI/KO mice (**m**–**p**), normalized to wild-type or control conditions. *, # and † denote groups that are significantly different by two-tailed Sidak (**b**–**d**) or Dunnett (**g**) post hoc testing of log-transformed data after one-way ANOVA (**b**–**d**,**g**), Holm–Sidak corrected multiple *t*-tests (**j**–**l**) or multiple *t*-tests after log transformation (**m**–**p**). For exact *P* values see Source Data. Source data

**Extended Data Fig. 6:**
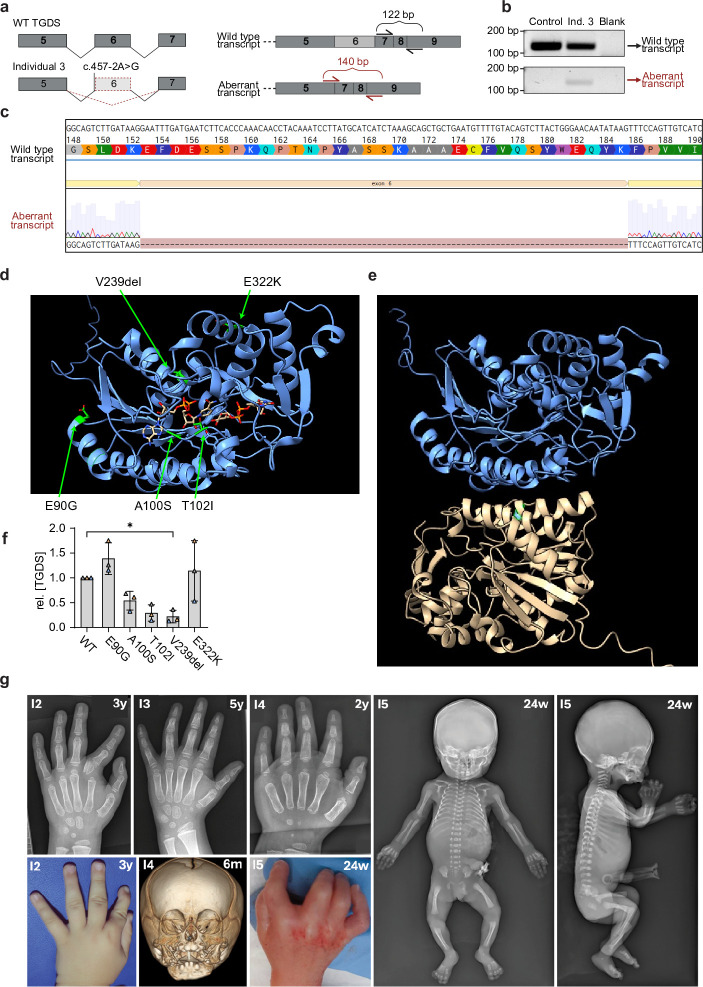
Characterization of clinical manifestations and *TGDS* variants in individuals with Catel-Manzke syndrome. **a-c**, Schematic representation of the *TGDS* variant in affected individual 3, abolishing the splice acceptor site of exon 6 (**a**; created in BioRender. Lyubenova, H. (2025) https://BioRender.com/l6uhom8), leading to a transcript lacking exon 6 as demonstrated by RT-PCR (**b**) and (**c**) sequencing, performed twice with the same result. **d-e**, Alphafold model of TGDS containing NAD^+^ and dTDP-glucose modeled based on the structure of prokaryotic dTDP-glucose dehydratases with Alphafill^73^ (**d**) or Alphafold model obtained with the Alphafold Multimer algorithm^74^ (**e**), predicting a dimeric protein. Amino acid variants observed in affected individuals are highlighted in green. V239, A100 and T102, as well as the region deleted in affected individual 3 are lining the catalytic pocket, whereas E90 and E322 are more peripheral. **f**, Quantification of TGDS expression normalized to GAPDH expression in U2OS cells transfected with the indicated variants containing a FLAG tag. Data are means ± SD from three independent experiments with two-tailed Dunnett *post-hoc* testing after one-way ANOVA. * denotes the only significant change (p-value 0.032). **g**, Photographs, radiographs and CT scans with 3D reconstruction of individuals (‘I’) with Catel-Manzke syndrome. Top row, hand radiographs of I2, I3 and I4. Lower row, hand photographs of I2 and I5 as well as cranial frontal CT scans with 3D reconstruction of I4. Right side, frontal and lateral whole body radiograph of I5 at 24 weeks of gestation, after pregnancy termination. Note radial deviation of the 2nd fingers in I2 and I5 as well as an accessory ossification center at the base of the 2nd proximal phalanx in I2 (Manzke dysostosis). Manzke dysostosis is a variable feature in Catel-Manzke syndrome. I3, I4 and I5 have clinodactyly of the 5th finger, I4 has additional clinodactyly of the 2nd, 3rd and 4th finger as well as distally enlarged metacarpals of the 2nd 3rd and 4th ray. Cranial CT scan of I4 shows asymmetric micrognathia. Whole body radiograph of I5 shows mildly shortened long bones, clinodactyly of the 5th fingers as well as micro- and retrognathia. I, individual; y, years; w, weeks; m, months. Source data

To our knowledge, no specific metabolic defect had been reported so far in fibroblasts from individuals with Catel–Manzke syndrome. If the function of TGDS was indeed the production of UDP-4-keto-6-deoxyglucose, levels of this metabolite should be reduced in fibroblasts of affected individuals. We obtained fibroblasts from three controls and five individuals with Catel–Manzke syndrome due to biallelic variants in *TGDS* (Fig. 4a, Extended Data Fig. 6 and Extended Data Table 1). Amounts of UDP-xylose and UDP-glucuronate were not systematically different from those in control fibroblasts (Fig. 4b,c). Although UDP-4-keto-6-deoxyglucose was below the detection limit of our analytical method, UDP-6-deoxyhexose levels were detectable in control cell lines and in more than 80% lower in fibroblasts from all affected individuals (Fig. 4d). The most parsimonious explanation for these observations is that fibroblasts carrying pathogenic variants in *TGDS* do not produce UDP-4-keto-6-deoxyglucose, which is the precursor for UDP-6-deoxyhexose. Furthermore, these experiments revealed that, similar to the situation in U2OS cells, UXS1 activity in fibroblasts can be maintained even at reduced concentrations of the rescue metabolite UDP-4-keto-6-deoxyglucose.

Some *TGDS* variants affect predicted cofactor binding sites whereas others do not (Fig. 4e and Extended Data Fig. 6d,e). When transiently transfected in U2OS cells, protein levels of *TGDS* variants were only significantly reduced for the p.Val239del variant (Fig. 4f and Extended Data Fig. 6f), indicating that reduced protein levels do not suffice to explain the loss of TGDS function. To explore whether they affected enzyme activity, we produced the corresponding recombinant proteins. All variants led to a significant decrease in activity at saturating substrate concentrations (Fig. 4g), whereas the *K*_M_ of the variants with residual activity was unchanged (Fig. 4h).

Overall, our experiments demonstrated that TGDS produces UDP-4-keto-6-deoxyglucose, which is required to overcome the functional inactivation of UXS1 in a cell-type-specific manner.

## Rescue metabolite loss in a mouse model

**Extended Data Fig. 7:**
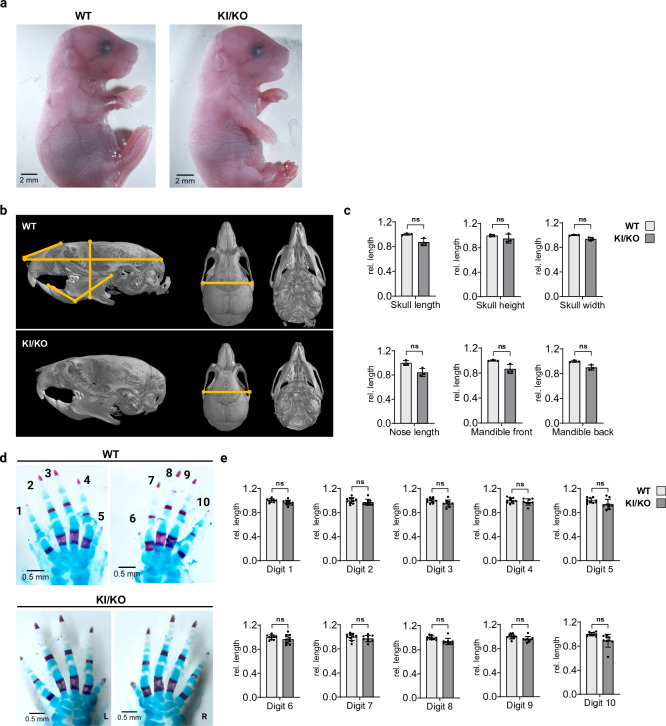
Characterization of *TGDS* KI/KO mice. **a**, Photographs of WT and KI/KO embryos at E18.5 show mildly shortened snouts and limbs in the mutants. Scale bar: 2 mm. **b**, µCT scans of 5-weeks-old WT and KI/KO mice. Measured parameters are shown in yellow. **c**, Quantification of skull measurements reveals tendencies similar to the ones observed in embryos, but they did not reach significance, likely due to smaller cohort sizes. Each measurement was normalized to the mean of the cohort of WT mice (littermates). Data are mean ± SD (n = 3). The differences between groups were analysed by two-tailed Welch’s t-test corrected for multiple testing according to Holm-Sidak. **d**, Skeletal preparations of front limb paws of WT and KI/KO embryos at E18.5 show normal digit morphology. **e**, Digit length was unaffected in the mutant animals. Each measurement was normalized to the mean of the WT littermates and data are mean ± SD (n = 8-10 for WT, and n = 8-9 for KI/KO). The differences between groups were analysed by two-tailed Welch’s t-test corrected for multiple testing by Holm-Sidak. L, left; R, right. Scale bar: 0.5 mm. For exact p-values see source data file. Source data

To explore whether the same pathogenic mechanism also occurred in vivo, we generated two different mouse lines using CRISPR–Cas9. One mouse line carried a heterozygous frameshift deletion–insertion, leading to a premature termination codon and the complete loss of protein function, and the other carried the heterozygous missense variant p.Ala100Ser, which is recurrent in individuals with Catel–Manzke syndrome (Fig. 4i). Initial studies demonstrated that complete inactivation of *Tgds* is lethal in early embryogenesis. We therefore generated compound heterozygous *Tgds*^A100S/−^ (KI/KO) mice by crossing the two mouse lines. We analysed the phenotype of *Tgds*-mutant mice at embryonic day 18.5 (E18.5) because we expected developmental defects. Using micro-computed tomography (µCT) scans of the skull, we observed a mild but significant reduction of the length of the skull, mandible and nasal bone (Fig. 4j and Extended Data Fig. 7a), without evidence of a cleft palate. Measurements of the long bones of the hindlimbs (femur, tibia and fibula) and forelimbs (humerus, radius and ulna) confirmed mild but significant shortening in mutant embryos (Fig. 4k,l). Comparable trends for facial skull changes were also observed in a cohort of mice analysed at five weeks of age (Extended Data Fig. 7b,c). In all instances, digit morphology was normal (Extended Data Fig. 7d,e). Together, the shortening of the long bones and the changes in the facial skeleton recapitulate the overall shorter stature and the facial changes in Catel–Manzke syndrome^3–6^.

Next, we explored the effect of *Tgds* deficiency on nucleotide sugar concentrations in the brain, liver, kidney and heart of mutant mice. The amount of UDP-4-keto-6-deoxyglucose—the product of Tgds—was more than 80% lower in brain, liver and kidney compared with wild-type mice, whereas it remained undetectable in the heart (Fig. 4m). Concomitantly, we also observed reduced amounts of UDP-6-deoxyhexose (Fig. 4n). On the basis of our observations with purified proteins and in cell lines, we expected that this might lead to an inhibition of UXS1 activity. Accordingly, we observed a decrease in UDP-xylose and an increase in the amount of UDP-glucuronate (Fig. 4o,p). Nonetheless, these changes were much less pronounced than in 293T and HAP1 knockout cell lines (Fig. 1c,d,h,i), potentially owing to some residual activity of the knockin allele. Furthermore, tissues consist of many different cell types. Therefore, metabolite changes in specific cell types might be masked owing to the presence of other cells that are not sensitive to the loss of *Tgds*.

Together, the KI/KO mouse model recapitulates important clinical aspects of Catel–Manzke syndrome, corroborates the biochemical function of TGDS and provides evidence that a functional UXS1 deficiency might underlie the clinical phenotype.

## TGDS deficiency affects specific glycans

The amount of UDP-xylose is strongly decreased, but not completely depleted, when we inactivate *TGDS* in HAP1 and 293T cells. To test whether these changes are functionally relevant, we analysed the production of two distinct glycans that are known to be dependent on the incorporation of xylose.

**Fig. 5:**
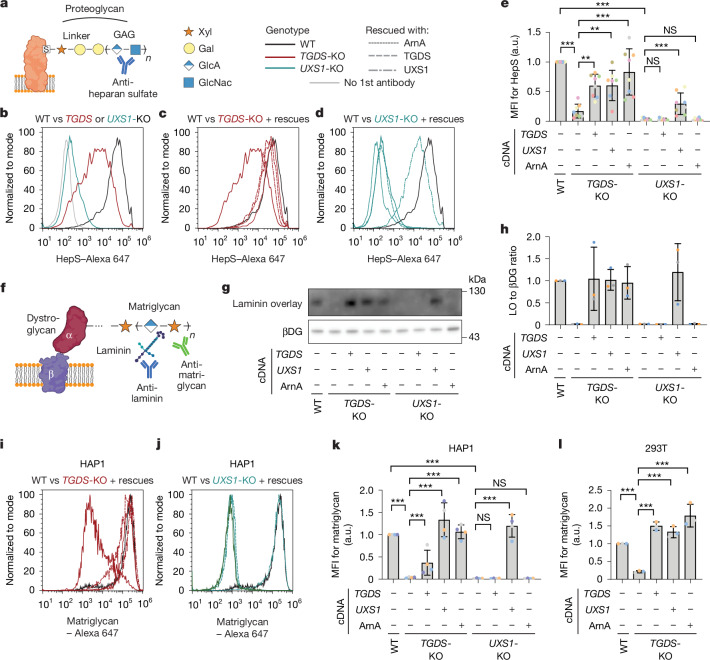
TGDS deficiency leads to reduced heparan sulfate formation and reduced glycosylation of α-dystroglycan. **a**, Schematic representation of the role of xylose in heparan sulfate. Xyl, xylose; Gal, galactose; GlcA, glucuronate; GlcNAc, *N*-acetylglucosamine. **b**–**e**, Representative histograms (**b**–**d**) and quantification of eight independent flow cytometry experiments (**e**) using an antibody against heparan sulfate in wild-type, *TGDS-*KO and *UXS1*-KO HAP1 cell lines and cell lines rescued with the indicated cDNAs. **f**, Schematic representation of the glycan of α-dystroglycan (matriglycan) and its detection by laminin overlay assay and flow cytometry. **g**, Representative laminin overlay analysis performed with samples from wild-type, *TGDS-*KO and *UXS1*-KO HAP1 cells. Western blot analysis for β-dystroglycan (βDG) on the same membrane is used as a control for dystroglycan abundance. For uncropped images see Supplementary Fig. 1. **h**, Quantification of three independent laminin overlay experiments. The signal of the laminin overlay (LO) was normalized to the β-dystroglycan western blot signal, and then to the wild type in each experiment. **i**–**l**, Representative histograms (**i**,**j**) and quantification of four (**k**) and three (**l**) flow cytometry experiments using an antibody against matriglycan in wild-type, *TGDS-*KO or *UXS1*-KO HAP1 (**i**–**k**) and 293T (**l**) cell lines as well as cell lines rescued with the indicated cDNAs. Data are mean ± s.d. from 8 (**e**), 3 (**h**), 4 (**k**) or 3 (**l**) independent experiments and are normalized to the mean of the value in wild-type cells. Paired two-tailed Sidak post hoc testing of log-transformed data after one-way ANOVA. MFI, mean fluorescent intensity; HepS, heparan sulfate. For exact *P* values see Source Data. Drawings in **a**,**f** Created in BioRender. Lyubenova, H. (2025) https://BioRender.com/hee241k. Source data

GAGs are long linear glycans that are linked to proteins via a tetrasaccharide linker with a xylose at its base^34^ (Fig. 5a). Many different GAGs exist, attached to many different proteins. We measured heparan sulfate via flow cytometry. Production of this glycan was clearly dependent on the presence of UDP-xylose, since genetic inactivation of *UXS1* led to a complete loss of the signal in HAP1 cells (Fig. 5b). Inactivation of *TGDS* in HAP1 cells also led to a reduction in heparan sulfate levels, indicating that the decrease in UDP-xylose was functionally relevant (Fig. 5b). Re-expression of the human proteins TGDS, UXS1 or the bacterial enzyme ArnA led to a partial recovery of heparan sulfate levels, providing support for our model that 4-keto sugar nucleotides are needed to reactivate UXS1 in cells (Fig. 5c–e).

α-dystroglycan is a cell surface protein that binds to several extracellular matrix proteins via a long series of glucuronate-xylose repeats^15^, called matriglycan. To assess the functionality of this glycan, we used flow cytometry with an antibody against matriglycan as well as a laminin overlay assay, in which membranes are first incubated with the extracellular matrix protein laminin followed by the detection of laminin-binding bands using a laminin antibody^35^ (Fig. 5f). We observed a robust signal in parental cells (Fig. 5g–l). By contrast, we detected no laminin overlay signal (Fig. 5g,h) and markedly reduced matriglycan signals (Fig. 5i–l) in *TGDS*-KO or *UXS1*-KO cells. This was owing to deficient glycosylation, since the amounts of β-dystroglycan, produced from the same protein precursor as α-dystroglycan, were unchanged (Fig. 5g). The phenotype was reversed in *TGDS-*KO cells when we restored the production of 4-keto sugar nucleotides by expressing the human enzyme TGDS or the bacterial enzyme ArnA (Fig. 5g–l). Of note, overexpression of UXS1 also rescued the phenotype, indicating that continuous production of ‘new’ UXS1 protein can overcome its inactivation (Fig. 5g–l). By contrast, the phenotype in cells lacking *UXS1* was only rescued when we overexpressed UXS1 (Fig. 5g–k), consistent with the notion that UDP-4-keto sugars act by reactivating a functionally inactivated UXS1.

Overall, these observations demonstrated that *TGDS* loss affects GAG synthesis and α-dystroglycan glycosylation in HAP1 cells, and that this deficiency can be rescued by supplying UDP-4-keto sugars.

## Discussion

This work reveals the molecular link between TGDS, UXS1, H6PD and Catel–Manzke syndrome. The identification of the enzymatic function of TGDS will help us understand the functional relevance of *TGDS* variants, facilitating a molecular diagnosis and informing genetic counselling.

UXS1 uses NAD^+^ as a cofactor that stays bound in its catalytic site and cycles between reduced and oxidized states during the conversion of UDP-glucuronate to its final product. However, the reaction intermediate occasionally escapes, leaving the enzyme inactive with NADH bound. Although replacing NADH with NAD^+^ could restore UXS1 activity, NAD^+^ is scarce in the endoplasmic reticulum (and likely in the Golgi apparatus) owing to H6PD activity in some cell types. TGDS activity produces low levels of UDP-4-keto-6-deoxyglucose, which restores NAD^+^ within the catalytic pocket of UXS1. The importance of this approach is underlined by the finding that all vertebrates have H6PD, UXS1 and TGDS, whereas insects have a UXS1 orthologue, but no H6PD or TGDS orthologues. Thus TGDS appears to be the price to pay to maintain both UXS1 and H6PD in the endoplasmic reticulum.

To date, H6PD has primarily been recognized for its role in maintaining a low NADP^+^/NADPH ratio within the endoplasmic reticulum, a requirement for 11β-hydroxysteroid dehydrogenase type 1 to convert cortisone into its biologically active form, cortisol^36,37^. However, it has also been shown—both in previous studies and confirmed in our own unpublished experiments—that H6PD acts almost as well with NAD^+^ as the cofactor as with NADP^+^^27,28^. The relevance of this activity is underscored by our findings that loss of H6PD bypasses the requirement for TGDS in supporting UXS1 function (Fig. 2g–k). Nevertheless, it remains unclear why NADH production in the endoplasmic reticulum would be advantageous. It is also unclear how the low NAD^+^/NADH ratio in the endoplasmic reticulum resulting from H6PD activity is maintained in the Golgi apparatus, where part of UXS1 resides. Changes in substrate availability, flux through the secretory pathway with concomitant transport of metabolites, and consumption of NADH might thereby affect the cell-type-dependent reliance on TGDS to maintain UXS1 function.

Life is dependent on a functional set of enzymes whose active sites can undergo various types of damage, including the formation of covalent adducts, damage of a cofactor or the loss of required modifications^38,39^. In some instances, such changes might have regulatory roles^40^, whereas in others, specialized enzymes may repair damage or replace cofactors^41–46^. However, recognizing the damage in a catalytic site represents a major challenge. In the case of TGDS, a mimic of the UXS1 reaction intermediate binds in the catalytic pocket and reactivates the cofactor using the enzyme’s native catalytic mechanism. This is reminiscent of the reactivation of glycolytic mutases, where a catalytic phosphate group is restored by metabolites that are related to their substrates^47,48^. Variants in the enzymes that produce these metabolites lead to clinical symptoms that do not seem to be linked to their enzyme-rescue function^49,50^. By contrast, *TGDS* deficiency leads to clinical symptoms resembling defects in GAG production, which is affected in cellular models of *TGDS* deficiency owing to the inactivation of UXS1.

Of note, the rescue metabolite is not only present at exceedingly low concentrations, but also corresponds to the dehydrated form of UDP-glucose, whose concentration is at least 1,000-fold higher. Similar signals are commonly generated from abundant metabolites during ionization in mass spectrometry and are often discarded as technical artefacts. The human metabolome, as detected by mass spectrometry, is much more complex than what would be necessary for canonical metabolic pathways. Although some of this complexity can be attributed to the formation of derivatives during mass spectrometry analysis^51^, our findings underscore that some of these presumptive artefacts may represent metabolites with important physiologic functions.

The molecular identities of most of the enzymes required for human intermediary metabolism are known, but the functions of some presumptive metabolic enzymes remain unknown^52^. Several of these proteins represent putative orthologues of bacterial metabolic enzymes that have no known utility in mammalian metabolism. It is tempting to speculate that some of these have been repurposed to produce non-canonical metabolites that help maintain the function of other enzymes. Our findings illustrate that for such ancillary metabolic functions rather slow enzymes, such as TGDS, can be sufficient and even desirable^39^.

Metabolic changes are pervasive in many diseases, but it is often unclear why these changes occur. When exploring the underlying causes and therapeutic approaches, the idea that enzymes can be functionally inactivated and then reactivated by rescue metabolites should be considered. Enzymes are continuously exposed to a variety of metabolites from endogenous metabolism, food and the microbiota. In some instances, the erroneous action of enzymes on specific metabolites might lead to their inactivation and clinical symptoms. A better understanding of these mechanisms might allow the development of approaches aiming to reactivate enzymes and improve treatment of rare and common diseases.

## Methods

### Human study participants

Affected individual 1 and parents of affected individuals 2, 3 and 4, and of fetus 5 provided their written consent for genetic testing, analysis of fibroblasts and publication of images. The study was performed according to the declaration of Helsinki and approved by the institutional Ethics Committees of Charité—Universitätsmedizin Berlin, Germany (EA2/101/18) and Necker Hospital Paris, France (IRB: 00011928, 2020-04-06). Information on sex, ethnicity and age of the study participants can be found in Extended Data Table 1. Blood, amniocyte and skin samples were obtained through standard procedures.

### Exome and Sanger sequencing

DNA from individuals 1, 2, 3 and 4 and parents of individuals 2, 3, 4 and 5 was extracted from peripheral-blood lymphocytes, and from fetus 5 and 6 from uncultured amniocytes according to standard protocols. Sanger sequencing of all exons of *TGDS* was applied to DNA of individual 2. Segregation analysis of detected *TGDS* variants using Sanger sequencing was performed on DNA of the parents of Individual 2, father of individual 3 and parents of individual 4. The primers used for Sanger sequencing of *TGDS* are listed in Supplementary Data Table 1. Exome sequencing was performed on DNA of individuals 1, 3, 4 and 5 as well as the mother of individual 3 and parents of individuals 4 and 5. Exome sequencing of individual 1 was previously described^2^. The technical approach for exome sequencing of individuals 3^53^ and 4^54^ were previously described. For trio exome sequencing of individual 5, DNA was enriched using Agilent SureSelect DNA + SureSelect OneSeq 300 kb CNV Backbone + Human All Exon V7 capture, and paired-end sequenced on the Illumina platform (GenomeScan, Leiden, the Netherlands). The aim was to obtain 10 Giga base pairs per exome with a mapped fraction of 0.99. The average coverage of the exome is ~50×. Duplicate and non-unique reads were excluded. Data were demultiplexed with bcl2fastq Conversion Software from Illumina. Reads were mapped to the genome using the BWA-MEM algorithm (reference: http://bio-bwa.sourceforge.net/). Sequence variant detection was performed by the Genome Analysis Toolkit HaplotypeCaller (reference: http://www.broadinstitute.org/gatk/). The detected sequence variants (gene package prenatal, version 2, 26-2-2021 (https://www.erasmusmc.nl/genoomdiagnostiek)) were filtered and annotated with Alissa Interpret software (Agilent Technologies) on quality (read depth ≥10), minor allele frequency (≥0.1% in 200 alleles in dbSNP, ESP6500, the 1000 Genome project, GoNL or the ExAC database) and location (within an exon or first or last 10 bp of introns). Variants were further selected based on three inheritance models (de novo autosomal dominant, autosomal recessive and X-linked recessive) and classified using Alamut Visual (interactive Biosoftware, SOPHiA GENETICS) according to the American College of Medical Genetics and Genomics (ACMG) guideline for sequence variants interpretation^55^ and ClinGen Sequence Variant Interpretation (SVI) General Recommendation for Using ACMG Criteria (https://clinicalgenome.org/working-groups/sequence-variant-interpretation). These variant classification criteria were also applied on *TGDS* variants detected in the other individuals (Extended Data Table 1).

### Embryonic stem cell targeting and transgenic mouse strains

G4 mES cells were maintained as previously described^56^. The single guide RNA (sgRNA) targeting mouse *Tgds* exon 4 (NM_029578.3) was designed using http://crispr.mit.edu/guides/. Complementary sgRNA oligonucleotides were subsequently annealed, phosphorylated and cloned into the BbsI site of dephosphorylated pX459 pSpCas9(BB)-2A-Puro vector^57^ (Addgene; #62988). For knock-in of the pathogenic mutation *Tgds* c.298 G > T into mouse embryonic stem cells, single-stranded oligodeoxynucleotides (ssODN) (60 pMol) were designed with asymmetric homology arms (HA) and phosphorothioate (PS) bonds as previously described^58,59^. Transfection of mouse embryonic stem cells and further processing was performed as previously described^60^. Potential structural variant and knock-in embryonic stem cell clones were first identified by PCR detection using the same genotyping primers as for the animals later and subsequently confirmed by Sanger sequencing. Primer sequences can be found in Supplementary Data Table 1. Mutant animals were produced through tetraploid or diploid aggregation^61^.

### Mouse models

Mice were maintained by crossing with wild-type C57BL6/J mice. All mice were housed in a centrally controlled environment with a 12 h light and 12 h dark cycle, temperature of 20–22.2 °C, and humidity of 30–50%. Bedding, food and water were routinely changed. All animal procedures were conducted as approved by the local authorities (LAGeSo Berlin) under the license numbers G0247/13 and G0176/19. Ages and developmental stages of mice are indicated in the figure legends.

### Skeletal preparations

Mouse embryos at stage E18.5 were collected and stained for bone and cartilage markers as follows. Embryos were eviscerated and fixed in 100% ethanol overnight. Following fixation, cartilage was stained using Alcian Blue staining solution (150 mg l^−1^ Alcian Blue 8GX in 80% ethanol and 20% acetic acid) overnight. Then embryos were post-fixed and washed in 100% ethanol overnight. Samples were pre-cleared with 0.2% KOH for a day and bones were stained with Alizarin Red (50 mg l^−1^ Alizarin Red S in 0.2% KOH) until the desired colour had developed. Rinsing and clearing was done using low concentrations (0.2%) of KOH and stained embryos were stored in 25% glycerol and imaged using a Zeiss Discovery V12 microscope and Leica DFC420 digital camera.

### µCT analysis

E18.5 embryos were fixed and scanned in 70% ethanol using a SkyScan 1172 X-ray microtomography system (Bruker µCT) at 5-μm resolution. 3D model reconstruction was done with the Bruker Micro-CT image analysis software NRecon and CTVOX.

### Cell culture

*HAP1 cells* were obtained from Horizon and cultured at 37 °C, 5% CO_2_ in IMDM (Biowest) supplemented with 10% FBS (Cytiva) and antibiotics (100 μg ml^−1^) penicillin streptomycin (Biowest). 293T and HCT116 cells were obtained from Eric Fearon (University of Michigan, MI, USA) and U2OS cell lines were obtained from Anabelle Decottignies (UCLouvain, Brussels, Belgium). 293T, HCT116, U2OS and human fibroblasts described in the present study were cultured at 37 °C, 5% CO_2_ in DMEM (Biowest) supplemented with 10% FBS (Cytiva), antibiotics (100 μg ml^−1^) penicillin streptomycin (Biowest) and 5% Ultraglutamine (Biowest). Cell lines were not further identified but were tested regularly for Mycoplasma.

### Generation of plasmids

Primers to generate plasmids are listed in Supplementary Data Table 1, and plasmids are listed in Supplementary Data Table 2. We used lentiviral expression constructs based on the plasmid pLVX-PURO (Clontech). In these constructs, expression is driven by the SV40 promoter (pUB82), the CMV promoter (pUB83) and the EF1α promoter (pUB81) (details available upon request).

We amplified the *TGDS* open reading frame (ORF) by PCR from a sequence-verified cDNA clone (Horizon discovery MGC clone Id 5175390) using the primers hTGDS_s_NheI and hTGDS_as_Acc65I. The resulting product was digested using the restriction enzymes NheI and Acc65I and inserted in the plasmids pUB82 and pUB83 using the restriction sites XbaI and BsrGI giving rise to the lentiviral plasmid pSP19 and pSP20 respectively. Subsequently, the *TGDS* open reading frame was transferred from pSP19 into the vector pUB81 via the restriction sites BamHI and EcoRI, leading to the plasmid pJJ45.

To generate plasmids carrying variants, *TGDS* (NM_014305.4) was amplified from cDNA and subcloned into pCMV6-Entry mammalian vector with and without C-terminal Myc-DDK tag. The variants were introduced using site-directed mutagenesis with Kapa Hotstart HiFi (Roche) and In-Fusion cloning kit (Clontech, Takara).

We amplified the ArnA ORF from an the K12 *E. coli* strain using the ecArnA_s_BamHI and ecArnA_as_BsrGI. The resulting PCR product was digested using the restriction enzymes BamHI and BsrGI, and inserted into the corresponding sites in the vector pUB82, resulting in the plasmid pJG406.

We amplified the *B. cinerea* UG46DH ORF from a geneblock (IDT) optimized for *E. coli* codon usage using the primers bfUG46DH_s_BamHI and bfUG46DH_as_EcorI. The sequence is based on the clone XM_001554921.2^30^. The resulting PCR product was digested and inserted into the plasmid pUB83 using the restriction enzymes BamHI and EcoRI, producing the plasmid pJJ43.

We amplified the human *UXS1* ORF from a sequence-verified cDNA clone (Horizon, Id 3843312), using the primers hUXS1_s_NheI and hUXS1_as_BsrGI. The resulting PCR product was digested using the restriction enzymes NheI and BsrGI and inserted in the BsrGI and XbaI sites in the plasmid pUB81 producing the plasmid pSP54.

The bacterial expression vector for TGDS, pJJ60, was generated in several steps using the open reading frame from pSP19, yielding a final construct with a N-terminal hexahistidine-tag fused to amino acid 15 of TGDS in the plasmid pET28a (Merck), via a PCR product with the primers hTGDS_s_G15 and hTGDS_as_XhoI (details and map available upon request). The same primers were used to transfer the open reading frames containing variants observed in affected individuals from the eukaryotic into the bacterial expression vector.

To generate the bacterial expression vector for ArnA, its ORF was amplified from K12 *E. coli* genomic DNA using primers ecArnA_s_NdeI and ecArnA_as_SacI. The resulting PCR product was digested with NdeI and SacI and inserted into the corresponding site in the plasmid pET28a, yielding the plasmid pJG413.

To generate a bacterial expression vector for *B. cinerea* UG46DH, its ORF was amplified from a geneblock (see above) using the primers bfUG46DH_s_NcoI and bfUG46DH_as_XhoI, and inserted into the corresponding sites in the plasmid pET28a, yielding the plasmid pJJ40.

To generate an expression vector for H6PD, its open reading framing was amplified from a sequence-verified clone (Horizon Discovery Ltd. Clone ID MHS 6278 202808130) using primers hH6PD_s_MluI and hH6PD_rev_EcoRI, and inserted into the corresponding sites of the plasmid pUB81 yielding the plasmid pIG425^62^. For transient transfection in mammalian cells, *H6PD* was amplified by PCR adding a V5 tag using Kapa Hotstart HiFi (Roche) and subcloned into pCMV6-Entry vector using In-Fusion cloning kit (Clontech, Takara).

CRISPR–Cas9 constructs to generate knockout cell lines were produced by ligating annealed oligonucleotides into the BbsI site of the vector pX459 or pX458 (Addgene #48138)^63^, as previously described (see Supplementary Data Table 1)^57,63^, or into the vector pLenticrispr V2.0 (Addgene #52961)^64^.

### Generation of knockout cell lines

To generate knockout cell lines, cells were transfected in 6-well plates at 70% confluence with 2 µg of the CRISPR–Cas9 guide RNA expression plasmids and 4 µl Lipofectamine 2000 following the manufacturer’s instructions (Life Technologies). Transfected cells were either transiently selected with puromycin (for pX459 constructs) or selected by flow cytometry sorting gating for GFP fluorescence on a FACSAria III flow cytometer (for pX458 constructs). Clonal populations were grown out and analysed by Sanger sequencing. To generate polyclonal knockout cell lines, recombinant lentiviruses driving expression of both CRISPR–Cas9 and specific guide RNAs were generated as described below.

### Lentiviral transduction

To produce recombinant lentiviruses, we transiently transfected 293T cells with lentiviral vectors and second generation packaging plasmids psPAX2 and pMD2.G (kind gifts from D. Trono, Addgene #12260 and #12259) using calcium phosphate precipitation^65^. The culture medium was changed 6 h after transfection, and recombinant viruses were recovered in the culture supernatant 24 h later. The virus-containing supernatant was then incubated with target cells in the presence of 4 μg ml^−1^ polybrene (Sigma). Infected cells were selected 24 h later for 4 days with puromycin (Thermofisher).

### Preparation of metabolite extracts for LC–MS analysis

For metabolomic analyses, non-fibroblast cell lines were plated in 6-well plates at 350,000 cells per well (500,000 for HAP1 cells). Lysates were obtained after quenching of metabolism as described before^66^. In brief, after one rapid wash with ice-cold water, culture plates were placed on liquid nitrogen for 5 s and then transferred onto dry ice. Cells were scraped after addition of 250 μl of a solution consisting of 90% methanol (Biosolve) and 10% chloroform, and lysates were transferred into microcentrifuge tubes. After centrifugation for 10 min at 4 °C and 27,000*g*, the supernatant was recovered, dried in a SpeedVac vacuum concentrating system (Life technologies) and resuspended in 30 μl of water before a final centrifugation of 10 min at 4 °C and 27,000*g*. Twenty microlitres of supernatant were transferred into LC–MS vials (Verex Vial, 9 mm Screw from Phenomenex) before analysis.

Fibroblasts were cultured in 10 cm dishes until 90% confluence. Cells were washed twice with 10 ml of ice-cold water per plate, and recovered using a cell scraper after addition of 500 μl of 0.5 M perchloric acid. Two plates were pooled into one tube followed by centrifugation at 27,000*g* at 4 °C for 10 min. The supernatant was recovered and transferred into a microcentrifuge tube. 100 µl of potassium carbonate was added to neutralize the solution. Samples were then spun down at 27,000*g*, 4 °C for 5 min and the supernatant was purified via solid phase extraction (SPE).

Mice were sacrificed at 8 months of age. Brain, heart, liver and kidney were obtained and immediately frozen in liquid nitrogen. Samples were pulverized with a mortar and pestle in liquid nitrogen. Approximately 50 mg of tissue powder was homogenized in a refrigerated Precellys bead beater (Bertin instruments) 6 times for 10 s at 10,000 oscillations per min in reinforced 2 ml tubes containing 500 µl of 0.5 M perchloric acid as well as seven 1.4 mm ceramic beads (Omni). After homogenization and centrifugation at 27,000*g* at 4 °C for 10 min, the supernatant was recovered and transferred into a microcentrifuge tube. Forty microlitres of potassium carbonate was added to neutralize the pH of the solution. The samples were then spun down at 27,000*g*, 4 °C for 5 min, and then purified via SPE.

The supernatants from neutralized perchloric acid extracts were loaded onto SPE columns (250 mg Supelclean ENVI-Carb SPE Tube from Supelco) pre-equilibrated by successive addition of 600 µl of 60% acetonitrile, 400 µl of 0.3% formic acid, and 3 ml of water. After sample application, the column was washed with 3 ml of water and 3 ml of acetonitrile. At all steps, the liquid was aspirated through the column via a Vac-Man Laboratory Vacuum Manifold from Promega. Metabolites were eluted into a microcentrifuge tube by addition of 1 ml 60% acetonitrile/0.3% formic acid. The eluate was dried in a SpeedVac vacuum concentrating system (Life Technologies) and resuspended in 25 μl of water before a final centrifugation of 10 min at 4 °C and 27,000*g*. Twenty microlitres of supernatant were transferred into LC–MS vials for analysis.

### LC–MS analysis

Analyses by LC–MS were performed as previously described^67^ based on a previously described method^68^. In brief, 5 μl of sample were analysed with an Inertsil 3 μm particle ODS-4 column (150 ×2.1 mm; GL Biosciences) at a constant flow rate of 0.2 ml min^−1^ with an Agilent 1290 HPLC system. Mobile phase A consisted of 5 mM hexylamine (Sigma-Aldrich) adjusted to pH 6.3 with acetic acid (Biosolve BV) and phase B of 90% methanol (Biosolve BV)/10% 10 mM ammonium acetate (Biosolve BV). The mobile phase profile consisted of the following steps and linear gradients: 0–2 min at 0% B; 2–6 min from 0 to 20% B; 6–17 min from 20 to 31% B; 17–36 min from 31 to 60% B; 36–41 min from 60 to 100% B; 41–51 min at 100% B; 51–53 min from 100 to 0% B; 53–60 min at 0% B. For analysis of metabolite extracts obtained from fibroblasts and organs, MS acquisition was stopped between minutes 9 and 10 to avoid the peak resulting from the perchloric acid extraction.

When exploring the effect of H6PD inactivation on UDP-4-keto-6-deoxyglucose levels in 293T and HAP1 cell lines, we had to ensure that small increases were not masked by the background noise. For this reason, 4 million cells were plated in 10 cm dishes and metabolites were collected two days later via the protocol described for fibroblasts.

For the analysis of enzymatic reactions, a shorter gradient was used where the mobile phase profile consisted of the following steps and linear gradients: 0–2 min at 0% B; 2–6 min from 0 to 24% B; 6–13 min from 24 to 31% B; 13–21 min from 31 to 50% B; 21–22 min from 50 to 100% B; 22–29 min at 100% B; 29–30 min from 100 to 0% B; 30–37 min at 0% B.

Analytes were identified with an Agilent 6550 ion funnel mass spectrometer operated in negative mode with an electrospray ionization (ESI) source and the following settings: ESI spray voltage 3500 V, sheath gas 350 °C at 11 l/min, nebulizer pressure 35 psig and drying gas 200 °C at 14 l min^−1^. An *m*/*z* range from 70 to 1,200 was acquired with a frequency of 1 per second by adding 8,122 transients. Compound identification was based on their exact mass (<5 ppm) and retention time compared to standards, obtained from Sigma-Aldrich (UDP-GlcNAc U4375, UDP-GlcA U6751, UDP-Glc U4625, CMP sialic acid 233264, GDP-mannose G5131) or Carbosource Services at the Complex Carbohydrate Research Center of the University of Georgia (UDP-Xyl, UDP-Ara). UDP-4k6dG and UDP-6dH were synthesized by ArnA or UG46DH as described below. CDP-ribitol was synthesized as described before^69^. The area under the curve of extracted-ion chromatograms of the [M-H]- forms were integrated using MassHunter software (Agilent), and normalized to the mean of the areas obtained for a series of 150 other metabolites (total ion current).

### Protein purification

Expression plasmids for human UXS1 were transformed into the BL21 Rosetta strain (Merck) using electroporation. A pool of >10 colonies was used to inoculate a 5 ml culture in Lysogeny broth (LB) containing 30 μg ml^−1^ kanamycin, and incubated overnight at 37 °C, shaking at 200 rpm. The preculture was added to 500 ml LB. Once this culture reached an optical density of 0.5 at 600 nm, we added 1 mM isopropyl-β-d-thiogalactoside (IPTG), and the culture was incubated overnight at 20 °C. Bacteria were collected by a 20 min centrifugation at 6,000*g* and 4 °C. Bacterial pellets were stored at −20 °C until purification. Cell pellets were resuspended in 25 ml of lysis buffer containing 50 mM HEPES pH 7.5, 500 mM NaCl, 5 mM imidazole, 5% glycerol and protease inhibitors (*p*-toluenesulfonyl fluoride (TSF) at 1 mM, leupeptin at 1 µg ml^−1^, and antipain at 1 µg ml^−1^) and lysed using a French Press (Glen Mills). The lysate was then centrifuged for 20 min at 20,000*g* and 4 °C. The supernatant was incubated with 1 ml Ni-NTA beads (Cytiva) for 10 min in a 15 ml tube at 4 °C on a rotative device. Beads were collected by centrifugation for 10 min at 400*g* and 4 °C, and resuspended in 3 ml of lysis buffer. The slurry was added in a 2 ml disposable Column (Pierce) and washed with 15 ml of lysis buffer supplemented with 30 mM imidazole, followed by 5 ml of lysis buffer supplemented with 100 mM of imidazole. Protein was eluted using 5 ml of lysis buffer containing 250 mM imidazole. Fractions containing the protein of interest were pooled, buffer-exchanged using a G25 Sepharose column (Cytiva PD-10) following the manufacturer’s protocol with a buffer containing 20 mM triethanolamine, 250 mM NaCl and 1 mM DTT, and stored at −80 °C. We obtained approximately 5 mg of protein per liter of culture.

Production of human TGDS and its mutants was performed using a similar protocol as for UXS1 with the following differences: After induction with IPTG, cultures were incubated overnight at 20 °C. We used a lysis buffer containing HEPES pH 7.4, 300 mM KCl, 1 mg ml^−1^ lysozyme (Roche), 10 mM imidazole, 3 mM β-mercaptoethanol, 10% glycerol, protease inhibitors (TSF at 2 mM, leupeptin at 1 µg ml^−1^, and antipain at 1 µg ml^−1^), and 5 µM NAD^+^, and lysed by French Press. The lysate was clarified by centrifugation for 20 min at 20,000*g* and 4 °C. We used 1 ml of a 50% HisPur Ni-NTA matrix slurry for purification. Washes were performed with five times 2 ml of lysis buffer and the protein was eluted by resuspension in 2× 500 µl of lysis buffer containing 250 mM of imidazole, and 2× 500 µl of lysis buffer containing 500 mM imidazole.

Comparable results were obtained when protein purification was performed by using a liquid chromatography system (Akta, Cytiva) using a 1 ml HisTrap HP Ni-NTA column with a flow rate of 1 ml min^−1^. The column was equilibrated in buffer A (25 mM Hepes 7.4, 10% glycerol, 300 mM KCl, 3 mM β-mercaptoethanol, 10 mM imidazole and 5 µM NAD^+^). After sample application, the column was washed with 20 column volumes of 94% buffer A and 6% buffer B (50 mM Hepes 7.4, 10% glycerol, 200 mM KCl, 5 mM β-mercaptoethanol, 500 mM imidazole, 0.2% sodium dodecyl maltoside, 1 mM TCEP) followed by a gradient to 100% over the course of 17 column volumes. We obtained approximately 0.6 mg of purified protein per liter of culture.

Production of ArnA was performed as described for human UXS1 with the following differences: the lysis buffer consisted of 100 mM HEPES pH 7.5, 150 mM KCl, 1 mg ml^−1^ lysozyme (Roche), 5 mM MgCl_2_, 5 mM β-mercaptoethanol, 10% glycerol and 1 mM of the protease inhibitor phenylmethylsulfonyl fluoride (PMSF). Lysis was performed by a freeze-thaw cycle in liquid nitrogen. After incubation for 15 min on ice, the concentration of KCl was brought to 500 mM and the lysate was sonicated. The lysate was clarified by centrifugation for 20 min at 20,000*g* and 4 °C, followed by purification with a liquid chromatography system (Akta, Cytiva) using a 1 ml HisTrap HP Ni-NTA column with a flow rate of 1 ml min^−1^. The column was equilibrated in buffer A (100 mM Hepes 7.4, 10% glycerol, 500 mM KCl, 5 mM MgCl_2_, 5 mM β-mercaptoethanol). After sample application, the column was washed with 20 column volumes of 94% buffer A and 6% buffer B (50 mM Hepes 7.4, 10% glycerol, 200 mM KCl, 5 mM β-mercaptoethanol, 300 mM imidazole) followed by a gradient to 100% over the course of 20 column volumes. We obtained more than 50 mg of purified protein per liter of culture.

Purification of *B. cinerea* UG46DH was performed as described for UXS1 with the following differences. Expression was induced overnight at 20 °C. The lysis buffer consisted of 100 mM Tris-HCl 7.4, 150 mM NaCl, 1 mg ml^−1^ lysozyme (Roche), 1 mM EDTA, 10% glycerol. Lysis was performed by sonication and the clarified lysate was purified using a liquid chromatography system with a 1 ml HisTrap HP Ni-NTA column. The column was equilibrated in buffer A (50 mM sodium phosphate buffer pH 8, 300 mM NaCl, 5 mM β mercaptoethanol). After sample application, the column was washed with 20 column volumes of 96% buffer A with 4% buffer B (50 mM sodium phosphate pH 8, 300 mM NaCl, 5 mM β-mercaptoethanol, 300 mM imidazole). Protein was eluted in a gradient to 100% buffer B over 20 column volumes. Positive fractions were pooled and buffer-exchanged into 50 mM Tris pH 8, 150 mM NaCl, 10% glycerol, 1 mM DTT, and 10 µM NAD^+^. We obtained approximately 2.5 mg of purified protein per liter of culture.

### Experimental setup for enzymatic assays

To produce UDP-4-keto-6-deoxyglucose via *B. cinerea* UG46DH, we used 20 µl reactions containing 25 mM triethanolamine, 1 mM UDP-glucose, 1 mM MgCl_2_, 10 mM DTT, and 2 µM of enzyme. Reactions were incubated for 1 h at 30 °C, heated to 100 °C for 2 min and extracted with 40 µl of CHCl_3_, followed by centrifugation at 27,700*g* for 10 min at 4 °C.

To produce UDP-4-keto-6-deoxyglucose via TGDS, a reaction of 500 µl containing 25 mM triethanolamine, 1 mM UDP-glucose, 0.1% BSA, 1 mM MgCl_2_ and 0.53 µM purified enzyme was incubated for 23 h at 30 °C, followed by 5 min at 85 °C and a centrifugation at 27,700*g* for 10 min at 4 °C.

The activity of UXS1 was assessed in 20 µl reactions containing 25 mM triethanolamine, 1 mM UDP-glucuronate, 10 mM DTT, 0.1% BSA, and 2 µM purified enzyme. Reactions were incubated for 1 h at 30 °C, followed by deproteinization by addition of 20 µl of methanol and 40 µl of chloroform. The aqueous phase was collected and analysed by LC–MS.

Production of UDP-4-ketoxylose using ArnA was performed in 500 µl reactions containing 25 mM triethanolamine, 1 mM UDP-glucuronate, 10 mM DTT, 0.1% BSA, 20 µM NAD^+^, 5 mM sodium pyruvate, and 5.5 U ml^−1^ rabbit muscle LDH (Sigma) to reoxidize NADH to NAD^+^, and 21.8 µM of ArnA. Reactions were incubated for 6 h at 30 °C followed by a centrifugation using a centrifugal ultrafiltration device (Vivaspin 500, Sartorius) using the manufacturer’s protocol to obtain the reaction product in the flow through.

To inactivate recombinant UXS1 with sodium borohydride, the enzyme was incubated at 2 µM for 45 min on ice in the presence of 5 mM NaBH_4_ in a reaction containing 25 mM triethanolamine, 1 mM DTT, and 0.1% BSA. 1% acetone was added on ice for another 30 min to destroy borohydride. To assess activity, the enzyme was incubated with 1 mM UDP-glucuronate for 1 h at 30 °C, followed by extraction with 3 volumes of chloroform:methanol 2:1, recovery of the aqueaous phase, and analysis by LC–MS.

In reactivation experiments, we incubated 0.56 µM inactivated recombinant UXS1 with 0.875 µM of UDP-4-keto-6-deoxyglucose produced by TGDS, containing residual UDP-glucose. This corresponds to the estimated concentration in the endoplasmic reticulum and Golgi apparatus and represents approximately a 1.5-fold excess relative to the inactivated UXS1 enzyme. TGDS reactions contained residual UDP-glucose. To exclude a confounding effect of UDP-glucose, control reactions therefore contained 17.5 µM UDP-glucose.

In reactivation experiment using ArnAs product, we used 1.25 µM UDP-4-ketoxylose, corresponding approximately to a 2-fold excess of this metabolite relative to the inactivated UXS1 enzyme.

Given the higher levels of UDP-4-keto-6-deoxyglucose observed in UG46DH overexpressing cells, we used a 100-fold excess of the UG46DH product relative to UXS1. To exclude a confounding effect of residual UDP-glucose from the UG46DH reaction, the control reaction contained 500 µM UDP-glucose.

### Laminin overlay

Laminin overlay assays were performed as previously described^69^. In brief, cells were lysed in PBS containing 1% Triton X-100 and centrifuged at 27,700*g* for 10 min at 4 °C. Supernatants were incubated under gentle rotation for 16 h at 4 °C with WGA-Agarose beads (Vector Laboratories) using 100 μl of beads per 1400 μg of HAP1 cells protein with PBS 1 containing 0.1% Triton X-100. Beads were washed 3 times with 1 ml of PBS containing 0.1% Triton X-100, proteins were released by incubation for 10 min at 72 °C in the presence of reducing sample buffer and resolved on 3-8% Tris-acetate gels (Life Technologies) for 75 min at 130 V. After overnight transfer at 30 mA onto a PVDF membrane, membranes were blocked with laminin-binding buffer (LBB; 50 mM Tris-HCl pH 7.4, 150 mM NaCl, 1 mM MgCl_2_ and 1 mM CaCl_2_) containing 3% BSA (Sigma) for 1 h at room temperature and incubated overnight with 1.15 μg ml^−1^ Laminin-111 (Sigma, L2020) in LBB. After three 10 min washes with LBB, membranes were incubated for 2 h at room temperature with rabbit anti-laminin antibody (Sigma, L9393) diluted 1:1000 in LBB containing 3% BSA, washed another three times for 10 min with LBB, incubated for 1 h at room temperature with horseradish peroxidase-coupled donkey anti-rabbit IgG antibody (GE healthcare, NA934V) diluted 1:15,000 in LBB containing 3% BSA. The signal for β-dystroglycan obtained with mouse anti-β-dystroglycan antibody (clone 7D11, 33701, Santa-Cruz, 1:1,000) in the same membrane was used to normalize for overall abundance of dystroglycan. Chemiluminescent signals were detected using a Cytiva Amersham ImageQuant 800 western blot imaging systems. Uncropped images are shown in Supplementary Data Fig. 1.

### Western blot analysis

U2OS cells were seeded at 5 × 10^5^ cells per well in 6-well format, followed by transfection of 1 µg of plasmid using Lipofectamine 3000 (Invitrogen) and Opti-MEM (Gibco) according to the manufacturer’s instructions. After 48 h proteins were extracted in RIPA buffer (150 mM NaCl, 50 mM Tris, 5 mM EDTA, 1% Triton X-100, 0.25% desoxycholate, 5% SDS) containing protease inhibitors (cOmplete; Roche) and phosphatase inhibitors (PhosSTOP; Roche). Total protein concentration was determined using Pierce BCA assay (Thermo Scientific). Twenty micrograms of protein per lane was separated by SDS–PAGE (12%), transferred to nitrocellulose membrane and probed with primary antibodies. Immunoblot staining was performed for TGDS (rabbit anti-TGDS; HPA040857, Atlas, 1:1,000) and GAPDH (mouse anti-GAPDH; AM4300, ThermoFisher, 1:2,000). Membranes were incubated with IRDye anti-rabbit 800CW and anti-mouse 680RD secondary antibodies (926-32211 and 926-68070, Li-Cor Biosciences, 1:10,000). Signals were detected with OdysseyFc Imaging System and quantification was performed using Image Studio (Li-Cor Biosciences). The signal of TGDS was normalized to its GAPDH signal, and then to the wild type within each experiment. Data are presented as mean ± s.d. obtained in three independent experiments. *P < 0.05 in post hoc testing after one-way ANOVA corrected according to Dunnett.

Western blots for H6PD and β-actin were performed as described above, but using PVDF membrane (Immobilon P, Milipore), horseradish peroxidase-coupled secondary antibodies, chemiluminescence peroxidase substrate (Immobilon Western Blot reagent, Milipore) and a Cytiva IQ600 system. Mouse anti-H6PD (TA501257, Origene, 1:1,000) and anti-β-actin (A5441, Sigma, 1:5,000) were used as primary antibodies.

Uncropped images are shown in Supplementary Data Fig. 1.

### Immunofluorescence

U2OS cells were grown on glass coverslips overnight (1.5 × 10^5^ cells per well), followed by transfection of 1 µg of plasmid using Lipofectamine 3000 (Invitrogen) and Opti-MEM (Gibco) according to the manufacturer’s instructions. After 48 h, cells were fixed in cold methanol for 10 min at 4 °C, or for 10 min at room temperature with 4% paraformaldehyde in 1× PBS followed by permeabilization with 0,4% Triton X-100 in 1× PBS for 15 min at room temperature for the staining of TGDS and GM130. Immunofluorescence staining was performed overnight in PBS containing 3% BSA at 4 °C using the following antibodies: TGDS (rabbit anti-TGDS; HPA040857, Atlas, 1:500); PDIA1 (mouse anti-PDIA1 (P4HB); ab2792, Abcam, 1:200); GM130 (mouse anti-GM130; 610823, BD Transduction Laboratories, 1:500); FLAG (mouse anti-FLAG M2; F1804, Sigma, 1:500); CALR (rabbit anti-Calreticulin; ab92516, Abcam, 1:500); V5 (rabbit anti-V5; V8137, Sigma, 1:400); H6PD (mouse anti-H6PD; TA501257, OriGene, 1:100) and GIANTIN (rabbit anti-Giantin; 621352, BioLegend, 1:1,000). Secondary antibody staining was performed using anti-mouse IgG Alexa Fluor 555 (A21422, Invitrogen, 1:1000) and anti-rabbit IgG Alexa Fluor 488 (A21206, Invitrogen, 1:1,000) for 1 h in 1× PBS at room temperature. Coverslips were mounted in Fluoromount G (Invitrogen). Images were taken using either LSM700 or LSM980 with Airyscan 2 (Zeiss).

### Flow cytometry

The presence of heparan sulfate on the surface of cells was assessed by flow cytometry using the Heparan sulfate antibody HS10E4 antibody (H1890 USBIOlogical). Cells were washed once with PBS and incubated for 5 min with 1 ml of non-enzymatic cell dissociation solution (Sigma) at 37 °C until detached. Cells were recovered by sequential addition of 2 ml and 1 ml of PBS + 2% FCS, and washed twice with 1 ml of PBS, spinning at 500*g* for 3 min in between. Cells were then washed twice with 2 ml of PBS + 2% FCS, centrifuging at 1,000*g* for 3 min in between. For staining, cells were resuspended in 100 μl PBS + 2% FCS containing 1:200 anti heparan sulfate antibody (clone 10E4, H1890 USBIOlogical), and incubated for 60 min on ice. After washes with PBS containing 2% FCS, the cells were stained with a 1:50 dilution of Alexa Fluor 647 (AffiniPure Goat Anti-Mouse IgM, µ chain specific from Jacksonimmuno) in PBS containing 2% FCS for 40 min on ice. Cells were again washed twice and analysed using a Fortessa or FACSverse flow cytometer (BD biosciences). The same protocol was followed for the detection of α-dystroglycan (mouse IgM clone IIH6C4, 05-593, Sigma-Aldrich) with 1% BSA and 0.1% sodium azide in PBS.

### Statistical analyses

Investigators were blinded with regard to the genotype of the mice being analysed. No specific randomization was performed but each mouse had a certain probability to be wild-type, heterozygote or KO/KI. No blinding or randomization was performed for in vitro experiments. Mouse sex was not taken into consideration since Catel–Manzke syndrome affects both female and make individuals. Samples sizes for in vivo experiments were based on published studies with similar experimental design and phenotypic analyses. No specific sample size calculation was performed for in vitro experiments. Statistical analyses were performed in Prism 10 (GraphPad) and were two-tailed. Pairwise comparisons were performed using multiple *t*-tests correcting for multiple testing according to Holm–Sidak^70^. When more than two conditions were compared we performed a one-way ANOVA followed by post hoc testing corrected for multiple testing according to Dunnett (for comparisons with one control)^71^ and Sidak (for selected comparisons)^70^. When one condition lacked detectable signals, no statistical comparisons were performed with this condition, and this condition was not included in the ANOVA (Fig. 3h and Extended Data Figs. 2e,f,  4i–l and  5a–c). When indicated, testing was performed on log-transformed data, leading to comparable variances between sample groups. When results from several independent experiments were performed, the analyses were performed on the means obtained within each experiment (indicated as symbols with strong contrast), but values from individual experiments are presented in partially transparent symbols^72^. In this setting, paired tests were used. For craniofacial and finger measurements in animals a comparison between the two groups was performed using two-tailed unpaired *t*-test with Welch’s correction followed by correction for multiple testing according to Holm–Sidak. Figures were generated in GraphPad Prism and Adobe Illustrator, and illustrations were drawn with BioRender (https://www.biorender.com).

### Reporting summary

Further information on research design is available in the Nature Portfolio Reporting Summary linked to this article.

## Online content

Any methods, additional references, Nature Portfolio reporting summaries, source data, extended data, supplementary information, acknowledgements, peer review information; details of author contributions and competing interests; and statements of data and code availability are available at 10.1038/s41586-025-09397-x.

## Supplementary information

Supplementary Information This Supplementary Information file contains Supplementary Figure 1: Uncropped images of gels; Supplementary Figure 2: Example of the flow cytometry gating strategy; Supplementary Table 1: Primers and synthetic DNA sequences; and Supplementary Table 2: Plasmid list.

Reporting Summary

## Source data

Source Data Fig. 1

Source Data Fig. 2

Source Data Fig. 3

Source Data Fig. 4

Source Data Fig. 5

Source Data Extended Data Fig. 1

Source Data Extended Data Fig. 2

Source Data Extended Data Fig. 4

Source Data Extended Data Fig. 5

Source Data Extended Data Fig. 6

Source Data Extended Data Fig. 7

## Figures and Tables

**Extended Data Table 1:**
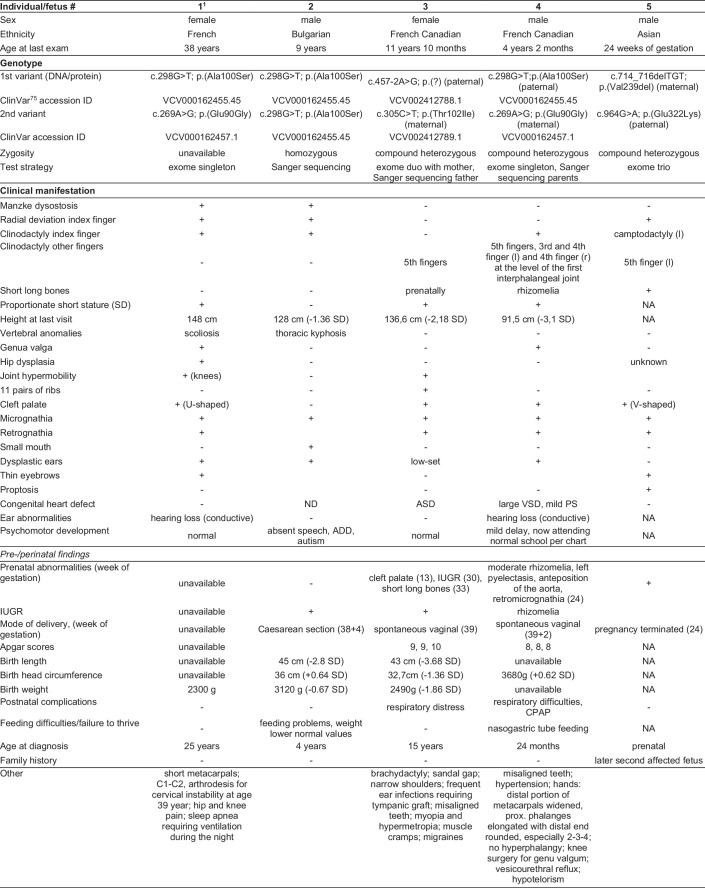
Genotype and phenotype of affected individuals with Catel-Manzke syndrome

The following clinical features were not detected in the present cohort: additional hyperphalangy, talipes, pectus deformity, laryngomalacia, highly arched palate/bifid uvula, widely spaced eyes, other organ malformations. L, left; r, right; NA, not applicable; ND, not determined; ADD, attention deficit disorder; ASD, atrial septal defect; VSD, ventricular septal defect; PS, pulmonal stenosis; IUGR, intrauterine growth retardation; CPAP, continuous positive airway pressure; SD, standard deviation; +, present; -, absent. Reference ^75^.

## Data Availability

All the data are contained in the paper or in the source data files. Source data are provided with this paper.
