# Supplementary material for: A missing enzyme-rescue metabolite as cause of a rare skeletal dysplasia
Source: Nature. 2025 Aug 20;646(8083):218–26. doi: 10.1038/s41586-025-09397-x (PMC12488480; doi:10.1038/s41586-025-09397-x)
Supplement: Supplementary file 1 — This Supplementary Information file contains Supplementary Figure 1: Uncropped images of gels; Supplementary Figure 2: Example of the flow cytometry gating strategy; Supplementary Table 1: Primers and synthetic DNA sequences; and Supplementary Table 2: Plasmid list. [file 41586_2025_9397_MOESM1_ESM.pdf]

nature portfolio

https://doi.org/10.1038/s41586-025-09397-x

Supplementary information

A missing enzyme-rescue metabolite as cause of a rare skeletal dysplasia

In the format provided by the authors and unedited

Supplementary Information

A missing enzyme-rescue metabolite as cause of a rare skeletal dysplasia

Jean Jacobs[†], Hristiana Lyubenova[†], Sven Potelle, Johannes Kopp, Isabelle Gerin, Wing Lee Chan, Miguel Rodriguez de los Santos, Wiebke Hülsemann, Martin A. Mensah, Valérie Cormier-Daire, Marieke Joosten, Hennie T. Bruggenwirth, Kyra E. Stuurman, Valancy Miranda, Philippe M. Campeau, Lars Wittler, Julie Graff, Stefan Mundlos, Daniel Ibrahim, Emile Van Schaftingen, Björn Fischer-Zirnsak, Uwe Kornak, Nadja Ehmke[*], Guido T. Bommer[*]

Table of contents

Supplementary Figures………..…………………..…….……………………………………...3

Supplementary Figure 1……………………………………………………………...3

Supplementary Figure 2……………………………………………………………...5

Supplementary Tables……………………………………………………………………...6

Supplementary Table 1……………………………………………………………… 6

Supplementary Table 2……………………………………………………………… 8

Supplementary Figure 1 | Uncropped images of gels for Fig. 4 and 5 as well as Extended Data Figures 2, 4 and 6.

Fig. 4f
Original same WB gel (reprobed)

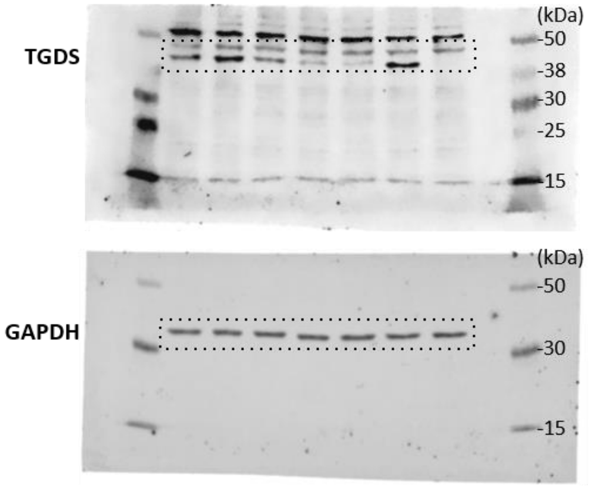

Fig. 5g
Original same WB gel (cut & different exposures)

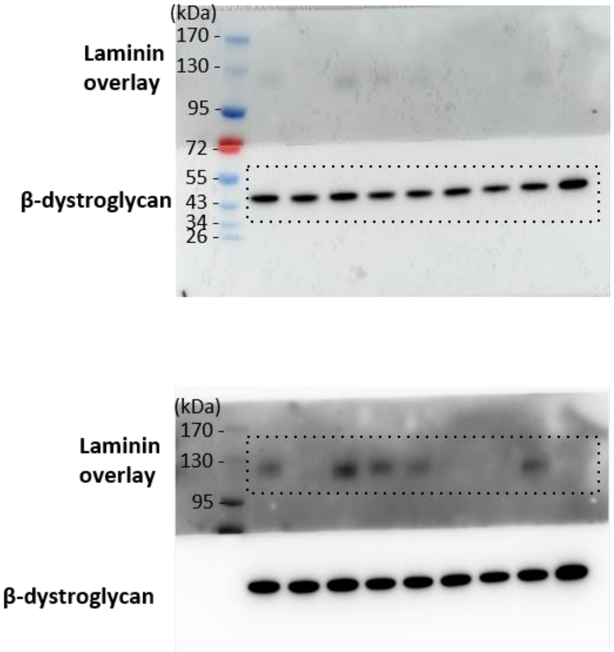

Ext. Data Fig. 2k
Original same WB gel (reprobed)

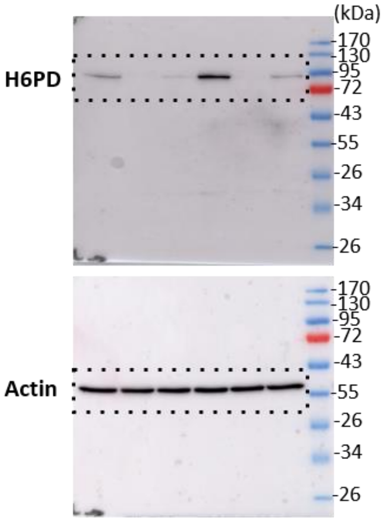

Ext. Data Fig. 2l
Original same WB gel (cut)

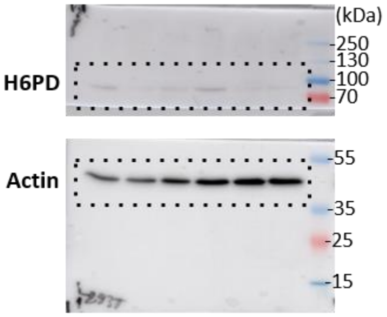

Ext. Data Fig. 2o
Original same WB gel (cut)

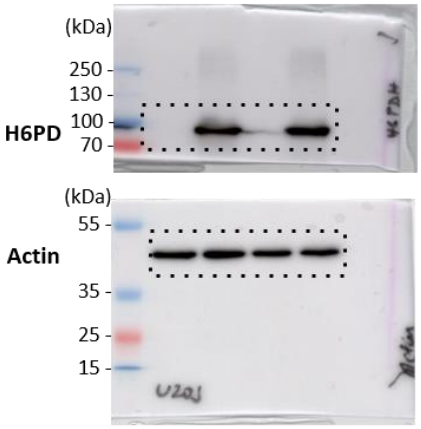

Ext. Data Fig. 2p
Original same WB gel (cut)

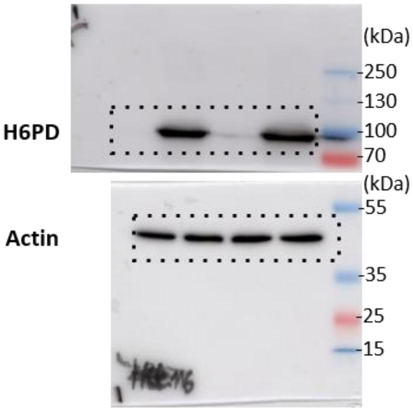

Ext. Data Fig. 4a
Coomassie-stained SDS-PAGE

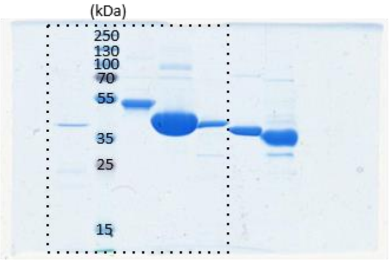

Ext. Data Fig. 6b
Agarose gel

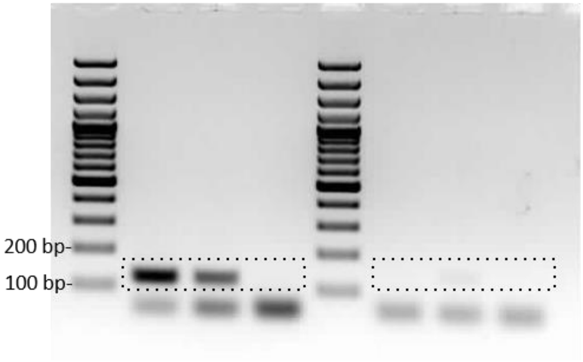

Supplementary Figure 2 | Example of the flow cytometry gating strategy for the experiments presented in Fig. 5.

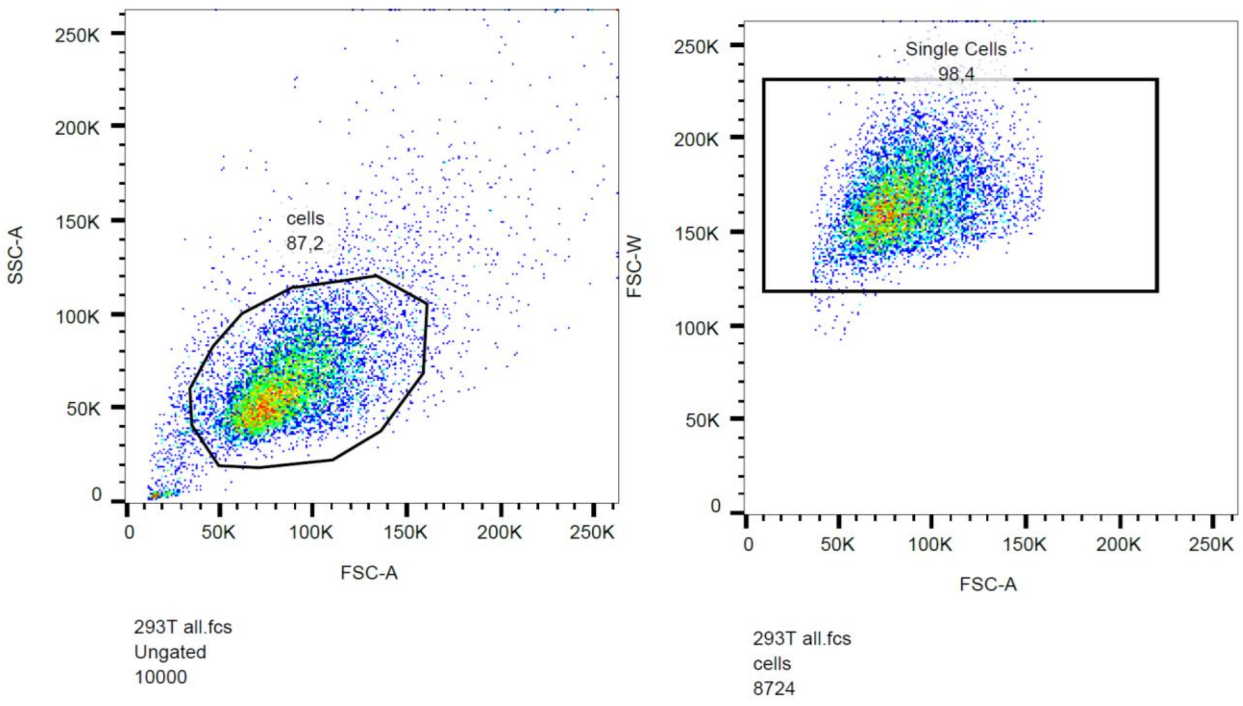

Supplementary Table 1 | Primers and synthetic DNA sequences used in this study.

Primers for cloning	Sequence
hTGDS_s_NheI	ATACATGCTAGCATGTCGGCGGCGTGT
hTGDS_as_Acc65I	GTATAAGGTACCTTATACCGGAAAGGG
ecArnA_s_BamHI	ATACATGGATCCACCATGAGCCAGCCTGCCTGCACCG
ecArnA_as_BsrGI	AAACGGTGTACATCATGATGGTTTATCCGTAAG
bfUG46DH_s_BamHI	TACTGCGGATCCATGGCCGCGAATGGTACTAC
bfUG46DH_as_EcorI	TACTGCGAATTCCTACGCCTCCACGGCCTGAGCCA
hUXS1_s_NheI	ATACATGCTAGCATGGTGAGCAAGGCGCTGCTGCGCC
hUXS1_as_BsrGI	GTATAATGTACATCAGCTGTGGCGAGTCCGTCCTTTC
hTGDS_s_G15	ATACATGCTAGCATGGGCTTTGCGAAGCGGGTCCT
hTGDS_as_XhoI	GCTAGTTATTGCTCAGCGG
ecArnA_s_NdeI	ATACATCATATGAGCCAGCCTGCCTGCACCG
ecArnA_as_SacI	AAACGGGAGCTCTCATGATGGTTTATCCGTAAG
bfUG46DH_s_NcoI	ATACGTCCATGGGCAGCAGCCATCATCACCATCACCACAGCAGCGGCGAGAATCTTTACTTTCAGTCCC ATATGGCCGCGAATGGTACTAC
bfUG46DH_as_XhoI	TACTGCCTCGAGCGCCTCCACGGCCTGAGCCA
hH6PD_s_MluI	ATACATACGCGTATGTGGAATATGCTCATAGTG
hH6PD_rev_EcoRI	TTATATGAATTCTCATCCCAGGAAGGCGTCGTAG
hTGDSvar_s_NcoI	ATACATCCATGGGCTTTGCGAAGCGGGTC
hTGDSvar_as_NotI	TTATATGCGGCCGCTACCGGAAAGGGTTCTAATGCCT
TGDS_p24_F	CGTCGACTGGATCCGGTACCGCCACCATGTCGGCGGCG
TGDS_p24_R	CGCGGCCGGCCGTTTATACCGGAAAGGGTTCTAATGCC
p50_A100S_F	ATTTTGCCTCACAAACACATGTAGATCTTTCATT
p50_A100S_R	TTTGTGAGGCAAAATGTAGTACTATATCTATT
p50_E90G_F	TGAAACAGGGAAAATAGATATAGTACTACATTTT
p50_E90G_R	ATTTTCCCTGTTTCAAAAAGCAGTTTCACA
p50_T102I_F	CGCACAAATACATGTAGATCTTTCATTCGTACG
p50_T102I_R	ACATGTATTTGTGCGGCAAAATGTAGTAC
p50_E322K_F	CTTGGAAAAAAGGAATAAAGAAAACAATTGAATG
p50_E322K_R	TTCCTTTTTTCCAAGGCACTTTAGGTCTCC
p50_V239del_F	ACTGATGTAGAAGCATTTCTCACTGTCCTC
p50_V239del_R	TGCTTCTACATCAGTAGCATAAAGGAAGTTTC
H6PD-p24 Fwd	CGTCGACTGGATCCGGTACCGCCACCATGTGGAATATGCTCA
H6PD-V5-p24 Rev	CGCGGCCGGCCGTTTACGTAGAATCGAGACCGAGGAGAGGGTTAGGGATAGGCTTACCTCCCAGGAAG GCGTCGT
**CRISPR/Cas9**	**Sequence**
hTGDS_exon5_s1	CACCGGATATGGTGGCAGTCTTGATA
hTGDS_exon5_as1	AAACTATCAAGACTGCCACCATATCC
hTGDS_exon5_s2	CACCGGTGTTTATGGCACTCACGTTT
hTGDS_exon5_as2	AAACAAACGTGAGTGCCATAAACACC

hUXS1_exon9_s1	CACCGCCTCAAAGTGAGGATTACTG
hUXS1_exon9_as1	AAACCAGTAATCCTCACTTTGAGGC
hUXS1_exon9_s2	CACCGAGGTCCTATTGGATTCACG
hUXS1_exon9_as2	AAACCGTGAATCCAATAGGACCTC
hH6PDH_exon2_s1	CACCGAGCTGTACCTGGATGAAGCG
hH6PDH_exon2_as1	AAACCGCTTCATCCAGGTACAGCTC
hH6PDH_exon4_Li1_s	CACCGGGTGAGGACCTCCGTCAGA
hH6PDH_exon4_Li1_as	AAACTCTGACGGAGGTCCTCACCC
**Mouse genotyping**	**Sequence**
Tgds_ES-2F	AAATACATGTGTTCTAAGATGTCAAAG
Tgds_ES-2R	ACAGAGCCTGATTCTTCCTG
Tgds_Int3_Fb	TGCAAGTAGGTGATAGAAGCCA
Tgds_Int4_Rb	GCTAAGTTTGATTCCTGAACCCT
Tgds_Int3_Seq-F	CTGGAGGGGGTGTTGAAGTA
Tgds_Int4_Seq-R	CAGAGCCTGATTCTTCCTGTCA
**Mouse generation**	**Sequence**
mTgds sgRNA	TTTGCTGCACAGACACATGT
ssODN	TGCTTAGTCAACCTATTCCTGAACTTGGGATTTATTCACTAGGGAAGGAAAATGCCAGTGGCAACCACAG<br>TATAAGGCAGTGCTTACCTACGTGCGTCTGCGAAGCAAAGTGCAGTACTATATCTATTTTCTCCACTTC
**Geneblock**	**Sequence**
UG46DH	ATGGCCGCGAATGGTACTACACCATCCTCCGCTAATGAGGAACAAAATAAATTTTTCGAGGACTTCGGAG<br>TGTGGAAAGAGGCTCCCATTTTAATCGGAAGCACTAAGTTTGAACCTTTGCCCGACGTTAAGAATATTAT<br>GATCACCGGCGGTGCTGGGTTTATTGCATGTTGGCTTGTGCGTCATTTGACTTTAACGTATCCCGACGCC<br>TATAATATTGTGAGCTTCGATAAGTTAGACTATTGTGCTAGTCTTAATAATACCCGTGCTCTTAACGATAAA<br>CGTAATTTTTCTTTCTATCACGGCGACATCACCAATCCCTCTGAAGTTGTTGACTGTTTAGAGCGTTATAA<br>CATCGACACAATCTTCCACTTTGCAGCCCAAAGCCATGTCGATCTGTCCTTCGGTAACTCCTATGCCTTC<br>ACACATACTAACGTATACGGCACTCACGTCCTTCTTGAGAGTGCCAAAAAAGTTGGTATTAAAAAATTTAT<br>CCACATCTCTACTGATGAGGTTTACGGTGAAGTAAAGGATGATGACGATGATTTATTGGAGACAAGTATC<br>TTAGCCCCGACAAATCCCTATGCAGCAAGCAAGGCGGCGGCTGAGATGCTTGTGCATAGTTATCAAAAG<br>TCCTTCAAGTTACCGGTGATGATTGTACGCTCGAACAACGTGTATGGCCCTCATCAATATCCTGAAAAGA<br>TCATTCCTAAATTCTCATGTCTTCTGCAACGTGGTCAGCCCGTCGTACTGCATGGAGACGGCACACCAAC<br>GCGCCGTTATTTATTCGCTGGTGATGCCGCAGATGCCTTCGACACTATCTTACACAAAGGAACTATCGGA<br>CAGATTTATAATGTGGGGAGTTACGACGAGATTTCCAACCTGACGTTATGCTCAAAACTTTTGACTTATCT<br>GGATATTCCACATAGTACTCAAGAGGAACTGCATAAATGGGTTAAACACACACAAGATCGTCCATTCAAT<br>GACCATCGCTACGCCGTTGACGGCACGAAACTTCGCCAACTGGGCTGGGATCAAAAGACGTCCTTCGAA<br>AATGGGATGGCTGTGACAGTTGACTGGTACAAGCGTTTTGGTGAGCGTTGGTGGGGGGACATTACTAAA<br>GTCTTAACTCCTTTTCCGACCGTGGCAGGCTCCAAAGTGGTGGGAGACGATAACAATACCGTGGAGGAA<br>TTGAAAGAAGAGATGGTAATCGACGCGGATGACAACATGATTTTAGGGAAAAAGCGTAAATTGAATGGG<br>GTCCCTTCTGGATTGGCTCAGGCCGTGGAGGCG
**Individual 3 Splice Variant PCR**	**Sequence**
hTgds_qPCR_e7-9_F	TCCAGTTGTCATCACAAGAAGCAGT
TGDS-e5-7_splice_F2	TGGCAGTCTTGATAAGTTTC
hTgds_qPCR_e7-9_R	GCCCTGACCCATGAATGCAACA

Supplementary Table 2 | Plasmids used in this study.

Plasmid n°	Name	Description
1	pUB82	pLVX-Puro plasmid with SV40 promoter
2	pSP19	Lentiviral vector expressing the TGDS under the promoter SV40
3	pUB81	pLVX-Puro plasmid with EF1a promoter
4	pJJ45	Lentiviral vector expressing the TGDS under the promoter EF1a
5	pJG406	Lentiviral vector expressing the ArnA under the promoter SV40
6	pUB83	pLVX-Puro plasmid with CMV promoter
7	pSP20	Lentiviral vector expressing the TGDS under the promoter CMV
8	pJJ43	Lentiviral vector expressing the UG46DH under the promoter CMV
9	pSP54	Lentiviral vector expressing the UXS1 under the promoter EF1a
10	pJG413	Bacterial vector for expression of N-terminally 6xHis-tagged ArnA
12	pJJ60	Bacterial vector for expression of N-terminally 6xHis-tagged Truncated TGDS (Starts at amino acid 15)
13	pJJ40	Bacterial vector for expression of N-terminally 6xHis-tagged UG46DH
16	pIG425	Lentiviral vector expressing the H6PD under the promoter EF1a
17	pJJ67	Bacterial vector for expression of N-terminally 6xHis-tagged Truncated TGDS containing the E90G variant (Starts at amino acid 15)
18	pJJ68	Bacterial vector for expression of N-terminally 6xHis-tagged Truncated TGDS containing the A100S variant (Starts at amino acid 15)
19	pJJ69	Bacterial vector for expression of N-terminally 6xHis-tagged Truncated TGDS containing the T102I variant (Starts at amino acid 15)
20	pJJ70	Bacterial vector for expression of N-terminally 6xHis-tagged Truncated TGDS containing the V239del variant (Starts at amino acid 15)
21	pJJ71	Bacterial vector for expression of N-terminally 6xHis-tagged Truncated TGDS containing the E322K variant (Starts at amino acid 15)
22	pJJ13	pX458 containing guide 1 against UXS1
23	pJJ14	pX458 containing guide 2 against UXS1
24	pIG420	pLenticrispr V2.0 containing guide 1 against H6PD
25	pIG422	pLenticrispr V2.0 containing guide 2 against H6PD
26	pSP02	pX459 containing guide 1 against TGDS
27	pSP13	pX459 containing guide 2 against TGDS
28	pHL24	pCMV_kozak-hTGDS_Myc-DDK-flag
29	pHL50	pCMV_kozak-hTGDS
30	pHL59	pCMV_kozak-hTGDS-E90G_Myc-DDK-flag
31	pHL60	pCMV_kozak-hTGDS-A100S_Myc-DDK-flag
32	pHL61	pCMV_kozak-hTGDS-T102I_Myc-DDK-flag
33	pHL62	pCMV_kozak-hTGDS-V239del_Myc-DDK-flag
34	pHL63	pCMV_kozak-hTGDS-E322K_Myc-DDK-flag
35	pHL58	pCMV_kozak-hH6PD-V5
