# Supplementary material for: A missing enzyme-rescue metabolite as cause of a rare skeletal dysplasia
Source: Nature. 2025 Aug 20;646(8083):218–26. doi: 10.1038/s41586-025-09397-x (PMC12488480; doi:10.1038/s41586-025-09397-x)
Supplement: Supplementary file 2 — Reporting Summary [file 41586_2025_9397_MOESM2_ESM.pdf]

Corresponding author(s): Nadja Ehmke & Guido T. Bommer

Last updated by author(s): Jul 4, 2025

Reporting Summary

Nature Portfolio wishes to improve the reproducibility of the work that we publish. This form provides structure for consistency and transparency in reporting. For further information on Nature Portfolio policies, see our Editorial Policies and the Editorial Policy Checklist.

Statistics

For all statistical analyses, confirm that the following items are present in the figure legend, table legend, main text, or Methods section.

n/a	Confirmed	
☐	☒	The exact sample size (*n*) for each experimental group/condition, given as a discrete number and unit of measurement
☐	☒	A statement on whether measurements were taken from distinct samples or whether the same sample was measured repeatedly
☐	☒	The statistical test(s) used AND whether they are one- or two-sided<br>*Only common tests should be described solely by name; describe more complex techniques in the Methods section.*
☒	☐	A description of all covariates tested
☐	☒	A description of any assumptions or corrections, such as tests of normality and adjustment for multiple comparisons
☐	☒	A full description of the statistical parameters including central tendency (e.g. means) or other basic estimates (e.g. regression coefficient) AND variation (e.g. standard deviation) or associated estimates of uncertainty (e.g. confidence intervals)
☐	☒	For null hypothesis testing, the test statistic (e.g. *F*, *t*, *r*) with confidence intervals, effect sizes, degrees of freedom and *P* value noted<br>*Give P values as exact values whenever suitable.*
☒	☐	For Bayesian analysis, information on the choice of priors and Markov chain Monte Carlo settings
☒	☐	For hierarchical and complex designs, identification of the appropriate level for tests and full reporting of outcomes
☒	☐	Estimates of effect sizes (e.g. Cohen's *d*, Pearson's *r*), indicating how they were calculated

Our web collection on statistics for biologists contains articles on many of the points above.

Software and code

Policy information about availability of computer code

Data collection	*Provide a description of all commercial, open source and custom code used to collect the data in this study, specifying the version used OR state that no software was used.*
Data analysis	Graphpad Prism v10,<br>Microsoft Office Suite<br>Adobe Illustrator 2025<br>BioRender (continuously updated online tool, no versions available)<br>Masshunter workstation Quantitative analysis (for TOF) software 10.2 agilent<br>Masshunter workstation Qualitative analysis software 10.0 agilent<br>SnapGene V 5.3<br>Benchling (continuously updated online tool, no versions available)<br>Alphafold Multimer algorithm<br>bcl2fastq Conversion Software from Illumina v2.20<br>BWA-MEM algorithm v0.7.17<br>Genome Analysis Toolkit HaplotypeCaller v3.7-0<br>Alissa Interpret software (Agilent Technologies) v5.3.4<br>Alamut Visual (interactive Biosoftware, SOPHiA GENETICS) v2.1.1<br>http://crispr.mit.edu/guides/, accession date: 02/02/2015<br>NRecon v1.7.4.6<br>CTVOX v3.3.0<br>ZEN v3.5 (blue edition)

ImageJ 1.52a
Image Studio v5.2

For manuscripts utilizing custom algorithms or software that are central to the research but not yet described in published literature, software must be made available to editors and reviewers. We strongly encourage code deposition in a community repository (e.g. GitHub). See the Nature Portfolio guidelines for submitting code & software for further information.

Data

Policy information about availability of data

All manuscripts must include a data availability statement. This statement should provide the following information, where applicable:

- Accession codes, unique identifiers, or web links for publicly available datasets
- A description of any restrictions on data availability
- For clinical datasets or third party data, please ensure that the statement adheres to our policy

We have included a data availability statement. The data presented in the figure are now presented in a source data file. There are no restrictions.

Research involving human participants, their data, or biological material

Policy information about studies with human participants or human data. See also policy information about sex, gender (identity/presentation), and sexual orientation and race, ethnicity and racism.

Reporting on sex and gender	We are investigating a rare disease that affects both male and female individuals. Given the limited availability of patient fibroblasts, data from female and male individuals were analyzed together.
Reporting on race, ethnicity, or other socially relevant groupings	Given the limited availability of patient fibroblasts, these factors were not taken into account. Yet, origins of patients are specified in their clinical description.
Population characteristics	The only defining feature of the groups was the presence or absence of pathogenic TGDS variants. As in any rare disease study, we cannot exclude confounding factors. This underlines the need to corroborate data in alternative experimental systems such as mouse models.<br>The ethnic background of the affected individuals was French, Bulgarian, French Canadian and Asian. Age ranged from prenatal (24th week of gestation) to 38 years. Two were female, three were male.
Recruitment	Catel-Manzke syndrome is a very rare disease. Therefore, affected individuals were identified in several different centers around the world without any preselection, except for the willingness to donate their fibroblasts. This approach is typical for rare monogenic diseases.
Ethics oversight	Written consent was obtained from the affected individuals and/or their healthy parents. The study was approved by the institutional Ethics Committees of Charité—Universitätsmedizin Berlin, Germany (EA2/101/18) and Necker Hospital Paris, France (IRB : 00011928, 2020-04-06).

Note that full information on the approval of the study protocol must also be provided in the manuscript.

Field-specific reporting

Please select the one below that is the best fit for your research. If you are not sure, read the appropriate sections before making your selection.

☒ Life sciences ☐ Behavioural & social sciences ☐ Ecological, evolutionary & environmental sciences

For a reference copy of the document with all sections, see nature.com/documents/nr-reporting-summary-flat.pdf

Life sciences study design

All studies must disclose on these points even when the disclosure is negative.

Sample size	Sample sizes were based on published studies using similar experimental designs and phenotypic analyses (PMID: 35704354; PMID: 28118357). The number of animals analyzed at each time point reflected both genotype availability, particularly the low-viability genotypes (KO/KO embryos), and the goal of ensuring consistent and interpretable phenotypic outcomes. The full penetrance of the phenotype in all KI/KO animals across developmental stages further supports the adequacy of the selected sample sizes.<br>No specific sample size calculation was performed for in vitro experiments.
Data exclusions	No data were excluded from the analyses. In some instances, the analysis of individual fingers was not possible when they were lost during the sample preparation. Likewise some individual samples were lost during sample preparation (--> see explanations in the source data file)
Replication	Studies with recombinant enzymes and in cells were performed in several independent experiments containing several independent samples. Wherever possible, we show these data as superplots highlighting both the mean within each independent experiment as well as individual

data points.

For mice, the situation is as follows:
Lethality: 3 litters with 9-12 embryos in total of which always only a single KO/KO embryo was detected – underdeveloped and partially digested.
At E18.5, a total of 20 embryos were analyzed (n=9 KI/KO and n=11 WT); all 9 KI/KO animals were affected.
At 5 weeks of age, a total of 6 animals were analyzed (n=3 KI/KO and n=3 WT); all 3 KI/KO animals were affected.
At 8 months of age, a total of 8 animals were analyzed (n=4 KI/KO and n=4 WT); all 4 KI/KO animals were affected.

Randomization

Investigators were blinded with regard to the genotype of the mice being analyzed. Thus, each mouse had a defined probability to be either wild type, heterozygote or KO/KI. No therapeutic intervention was performed.
For in vitro studies no randomization was performed. Yet, effects of knockout interventions were controlled by rescue experiments. No treatment was introduced that would warrant a randomization.

Blinding

Investigators were blinded with regard to the genotype of the mice being analysed. For in vitro studies, no blinding was performed.

Reporting for specific materials, systems and methods

We require information from authors about some types of materials, experimental systems and methods used in many studies. Here, indicate whether each material, system or method listed is relevant to your study. If you are not sure if a list item applies to your research, read the appropriate section before selecting a response.

Materials & experimental systems

n/a	Involved in the study	
☐	☒	Antibodies
☐	☒	Eukaryotic cell lines
☐	☐	Palaeontology and archaeology
☐	☒	Animals and other organisms
☒	☐	Clinical data
☐	☐	Dual use research of concern
☐	☐	Plants

Methods

n/a	Involved in the study	
☐	☐	ChIP-seq
☐	☒	Flow cytometry
☐	☐	MRI-based neuroimaging

Antibodies

Antibodies used

The following antibodies were used in this study: anti-TGDS (Atlas, HPA040857, polyclonal, lot R38059); anti-GAPDH (ThermoFisher, AM4300, clone 6C5, lot 2597762); anti-H6PD (Origene, TA501257, clone OTI2A7, lot W002); anti-PDIA1 (P4HB) (Abcam, ab2792, clone RL90, lot GR3225321-2); anti-GM130 (BD Transduction, 610823, clone 35/GM130 (RUO), lot 3200321); anti-FLAG (Sigma, F1804, clone M2, lot 0000375608); anti-Calreticulin (Abcam, ab92516, clone EPR3924, lot 1049597-20); anti-V5 (Sigma, V8137, polyclonal, lot #117M4821V); anti-Giantin (BioLegend, 621352, clone Poly23A01, lot B410237); anti-β-actin (Sigma, A5441, clone AC-15, lot 079M4799V); anti-HS (Heparan sulfate) (USBiological, H1890, clone 10E4); anti-laminin antibody (Sigma, L9393, polyclonal, lot 0000177605); anti-α-dystroglycan (Sigma-Aldrich, 05-593, clone IIH6C4, lot 3286105); and β-dystroglycan (Santa Cruz, sc-33701, clone 7D11, lot B1716).

Validation

Antibody validation was performed through various methods. TGDS showed no signal for the endogenous protein but did upon overexpression. GAPDH is published in Nat. Commun. (PMID: 32238925). H6PD was validated by knockdown in the manuscript and overlapping staining with V5-tagged H6PD protein, additionally it was published in Sci. Transl. Med. (PMID: 34039740). PDIA1 was published in Cell (PMID: 28984163) and further validated by colocalization with the ER marker Calreticulin. GM130 was published in Nat. Commun. (PMID: 31324769; 30478271; 28924207) and validated by colocalization with the Golgi marker Giantin. FLAG was published in Nat. Commun. (PMID: 25697406; 26215093). Calreticulin validation is supported by Nat. Commun. (PMID: 36241646; 36008380) and colocalized with PDIA1. V5 was published in Nat. Commun. (PMID: 29150614; 27193971) and colocalized with H6PD-V5. Giantin was published in PLoS One (PMID: 38848420) and colocalized with GM130. β-actin is published in Nat. Commun. (PMID: 31399589; 24300912). Heparan sulfate and laminin were validated by absence of signal in negative controls (UXS1 knockout). α- and β-dystroglycan are extensively validated in the literature (Nat. Commun., PMID: 27194101).

Eukaryotic cell lines

Policy information about cell lines and Sex and Gender in Research

Cell line source(s)

HEK293T and HCT116 cells were obtained from Eric Fearon (University of Michigan,MI, USA), and U2OS cell lines were obtained Anabelle Decottignies (UCLouvain, Brussels,Belgium). Commercially, HCT116 (CCL-247), U2OS (CCL-247), and 293T (CRL-3216) cells can be obtained from ATCC, and HAP1 cells can be obtained from Horizon Biodiscovery.

Authentication

Cell lines were not further authenticated.

Mycoplasma contamination	Cell lines were negative in a PCR-based mycoplasma assay.
Commonly misidentified lines (See ICLAC register)	The cell lines used in this study do not fall in this category.

Palaeontology and Archaeology

Specimen provenance	*Provide provenance information for specimens and describe permits that were obtained for the work (including the name of the issuing authority, the date of issue, and any identifying information). Permits should encompass collection and, where applicable, export.*
Specimen deposition	*Indicate where the specimens have been deposited to permit free access by other researchers.*
Dating methods	*If new dates are provided, describe how they were obtained (e.g. collection, storage, sample pretreatment and measurement), where they were obtained (i.e. lab name), the calibration program and the protocol for quality assurance OR state that no new dates are provided.*

☐ Tick this box to confirm that the raw and calibrated dates are available in the paper or in Supplementary Information.

Ethics oversight	*Identify the organization(s) that approved or provided guidance on the study protocol, OR state that no ethical approval or guidance was required and explain why not.*

Note that full information on the approval of the study protocol must also be provided in the manuscript.

Animals and other research organisms

Policy information about studies involving animals; ARRIVE guidelines recommended for reporting animal research, and Sex and Gender in Research

Laboratory animals	Mus musculus: C57/BL6J. We used animals at embryonic stages E10.5 (confirmation of lethality of KO/KO) and E18.5 (gross morphology imaging, µCT and skeletal preps) as well as at 5 weeks of age (µCT) and 8 months of age (LC-MS analysis of organ lysates).
Wild animals	We did not use any wild animals.
Reporting on sex	The disease under investigation affects both male and female individuals. Data from male and female mice were analyzed together.
Field-collected samples	We did not collect samples in the field
Ethics oversight	All animal procedures were conducted as approved by the local authorities (LAGeSo Berlin) under the license numbers 23 G0247/13 and G0176/19.

Note that full information on the approval of the study protocol must also be provided in the manuscript.

Dual use research of concern

Policy information about dual use research of concern

Hazards

Could the accidental, deliberate or reckless misuse of agents or technologies generated in the work, or the application of information presented in the manuscript, pose a threat to:

No	Yes	
☒	☐	Public health
☒	☐	National security
☒	☐	Crops and/or livestock
☒	☐	Ecosystems
☒	☐	Any other significant area

Experiments of concern

Does the work involve any of these experiments of concern:

No	Yes	
☒	☐	Demonstrate how to render a vaccine ineffective
☒	☐	Confer resistance to therapeutically useful antibiotics or antiviral agents
☒	☐	Enhance the virulence of a pathogen or render a nonpathogen virulent
☒	☐	Increase transmissibility of a pathogen
☒	☐	Alter the host range of a pathogen
☒	☐	Enable evasion of diagnostic/detection modalities
☒	☐	Enable the weaponization of a biological agent or toxin
☒	☐	Any other potentially harmful combination of experiments and agents

Plants

Seed stocks	*Report on the source of all seed stocks or other plant material used. If applicable, state the seed stock centre and catalogue number. If plant specimens were collected from the field, describe the collection location, date and sampling procedures.*
Novel plant genotypes	*Describe the methods by which all novel plant genotypes were produced. This includes those generated by transgenic approaches, gene editing, chemical/radiation-based mutagenesis and hybridization. For transgenic lines, describe the transformation method, the number of independent lines analyzed and the generation upon which experiments were performed. For gene-edited lines, describe the editor used, the endogenous sequence targeted for editing, the targeting guide RNA sequence (if applicable) and how the editor was applied.*
Authentication	*Describe any authentication procedures for each seed stock used or novel genotype generated. Describe any experiments used to assess the effect of a mutation and, where applicable, how potential secondary effects (e.g. second site T-DNA insertions, mosiacism, off-target gene editing) were examined.*

ChIP-seq

Data deposition

☐ Confirm that both raw and final processed data have been deposited in a public database such as GEO.

☐ Confirm that you have deposited or provided access to graph files (e.g. BED files) for the called peaks.

Data access links<br>*May remain private before publication.*	*For "Initial submission" or "Revised version" documents, provide reviewer access links. For your "Final submission" document, provide a link to the deposited data.*
Files in database submission	*Provide a list of all files available in the database submission.*
Genome browser session<br>(e.g. UCSC)	*Provide a link to an anonymized genome browser session for "Initial submission" and "Revised version" documents only, to enable peer review. Write "no longer applicable" for "Final submission" documents.*

Methodology

Replicates	*Describe the experimental replicates, specifying number, type and replicate agreement.*
Sequencing depth	*Describe the sequencing depth for each experiment, providing the total number of reads, uniquely mapped reads, length of reads and whether they were paired- or single-end.*
Antibodies	*Describe the antibodies used for the ChIP-seq experiments; as applicable, provide supplier name, catalog number, clone name, and lot number.*
Peak calling parameters	*Specify the command line program and parameters used for read mapping and peak calling, including the ChIP, control and index files used.*
Data quality	*Describe the methods used to ensure data quality in full detail, including how many peaks are at FDR 5% and above 5-fold enrichment.*
Software	*Describe the software used to collect and analyze the ChIP-seq data. For custom code that has been deposited into a community repository, provide accession details.*

Flow Cytometry

Plots

Confirm that:

☒ The axis labels state the marker and fluorochrome used (e.g. CD4-FITC).

☒ The axis scales are clearly visible. Include numbers along axes only for bottom left plot of group (a 'group' is an analysis of identical markers).

☒ All plots are contour plots with outliers or pseudocolor plots.

☒ A numerical value for number of cells or percentage (with statistics) is provided.

Methodology

Sample preparation	Cells were detached with Tryp-LE and stained with anti-HepS antibody followed by staining with a anti-IgM-AlexaFluor 647
Instrument	FACSverse
Software	Flowjo 10 for analysis
Cell population abundance	We did not quantify population abundances, but only geometric means of the intensities within a population..
Gating strategy	We only gated on life single cells.

☒ Tick this box to confirm that a figure exemplifying the gating strategy is provided in the Supplementary Information.

Magnetic resonance imaging

Experimental design

Design type	*Indicate task or resting state; event-related or block design.*
Design specifications	*Specify the number of blocks, trials or experimental units per session and/or subject, and specify the length of each trial or block (if trials are blocked) and interval between trials.*
Behavioral performance measures	*State number and/or type of variables recorded (e.g. correct button press, response time) and what statistics were used to establish that the subjects were performing the task as expected (e.g. mean, range, and/or standard deviation across subjects).*

Acquisition

Imaging type(s)	*Specify: functional, structural, diffusion, perfusion.*
Field strength	*Specify in Tesla*
Sequence & imaging parameters	*Specify the pulse sequence type (gradient echo, spin echo, etc.), imaging type (EPI, spiral, etc.), field of view, matrix size, slice thickness, orientation and TE/TR/flip angle.*
Area of acquisition	*State whether a whole brain scan was used OR define the area of acquisition, describing how the region was determined.*

Diffusion MRI ☐ Used ☐ Not used

Preprocessing

Preprocessing software	*Provide detail on software version and revision number and on specific parameters (model/functions, brain extraction, segmentation, smoothing kernel size, etc.).*
Normalization	*If data were normalized/standardized, describe the approach(es): specify linear or non-linear and define image types used for transformation OR indicate that data were not normalized and explain rationale for lack of normalization.*
Normalization template	*Describe the template used for normalization/transformation, specifying subject space or group standardized space (e.g. original Talairach, MNI305, ICBM152) OR indicate that the data were not normalized.*
Noise and artifact removal	*Describe your procedure(s) for artifact and structured noise removal, specifying motion parameters, tissue signals and physiological signals (heart rate, respiration).*
Volume censoring	*Define your software and/or method and criteria for volume censoring, and state the extent of such censoring.*

Statistical modeling & inference

Model type and settings

Specify type (mass univariate, multivariate, RSA, predictive, etc.) and describe essential details of the model at the first and second levels (e.g. fixed, random or mixed effects; drift or auto-correlation).

Effect(s) tested

Define precise effect in terms of the task or stimulus conditions instead of psychological concepts and indicate whether ANOVA or factorial designs were used.

Specify type of analysis: ☐ Whole brain ☐ ROI-based ☐ Both

Statistic type for inference

(See Eklund et al. 2016)

Specify voxel-wise or cluster-wise and report all relevant parameters for cluster-wise methods.

Correction

Describe the type of correction and how it is obtained for multiple comparisons (e.g. FWE, FDR, permutation or Monte Carlo).

Models & analysis

n/a	Involved in the study
☐	☐ Functional and/or effective connectivity
☐	☐ Graph analysis
☐	☐ Multivariate modeling or predictive analysis

Functional and/or effective connectivity

Report the measures of dependence used and the model details (e.g. Pearson correlation, partial correlation, mutual information).

Graph analysis

Report the dependent variable and connectivity measure, specifying weighted graph or binarized graph, subject- or group-level, and the global and/or node summaries used (e.g. clustering coefficient, efficiency, etc.).

Multivariate modeling and predictive analysis

Specify independent variables, features extraction and dimension reduction, model, training and evaluation metrics.
